# Supplemental nitrogen induces robust physiological and molecular adaptations by enhancing carbon metabolism in maize

**DOI:** 10.1007/s00709-025-02116-3

**Published:** 2025-09-26

**Authors:** Joseph N. Amoah, Claudia Keitel, Brent N. Kaiser

**Affiliations:** School of Life and Environmental Sciences, University of Sydney, 380 Werombi Road, Brownlow Hill, Camden, NSW 2570 Australia

**Keywords:** Assimilate partitioning, Diurnal sugar allocation, Nitrogen level supplementation, Nitrogen use efficiency, Maize adaptation

## Abstract

Plants depend on nitrogen (N) to support their growth, development, and essential metabolic activities. However, the mechanisms modulating the distribution of N assimilates under supplemental N (SN) condition is unknown. This study examines carbon (C) metabolism and spatial distribution in maize seedlings subjected to three N treatments (T1 to T3): T1, 1 mM NO₃⁻ (low N, LN); T2, supplementation of 1 mM NO₃⁻ with 2 mM NO₃⁻ (1 mM NO₃⁻ → 2 mM NO₃⁻, SN); and T3, 2 mM NO₃⁻ (medium N, MN). SN treatment induced significant physiological and molecular adaptations, such as enhanced growth and total biomass under fluctuating N conditions. SN-treated plants exhibited enhanced photosynthetic activity and significantly greater accumulation of soluble sugars, sucrose, and starch compared to those under LN and MN treatments. Activities of key C metabolism enzymes, such as sucrose phosphate synthase (SPS), sucrose synthase (SuSy) and invertases (INVs), starch synthase (SS), AGPase, α-amylase (AMY) and β-amylase (BAM) were significantly upregulated, supporting efficient C metabolism. Molecular analysis revealed transcriptional reprogramming under SN, marked by the upregulation of genes related to sucrose (*ZmSPS1*, *ZmSuSy1*, *ZmINVs*, *ZmSUT2*, *ZmSTP2*, *ZmSUC2* and *ZmSWEET14*) and starch (*ZmSS1*, *ZmAGPase1*, *ZmAMY1* and *ZmBAM1*) metabolism and transport. The spatial and diurnal analysis revealed dynamic C partitioning and adaptive regulation, with SN plants maintaining higher sucrose and starch levels in the leaves, sheath and roots. These findings highlight the robust plasticity of maize C metabolism under SN conditions and provide valuable insights into optimizing nitrogen use efficiency (NUE) for sustainable crop production. Future studies will focus on exploring these adaptive mechanisms across different maize genotypes and under field conditions to improve NUE and productivity in varying N environments.

**Supplementary Information:**

The online version contains supplementary material available at 10.1007/s00709-025-02116-3.

## Introduction

Nitrogen (N) is an essential macronutrient required for plant development, serving as a structural component of essential biomolecules such as nucleic acids, amino acids, and proteins. It also functions as a key signalling molecule, regulating various plant metabolic processes, including photosynthesis, seed regulation, responses to abiotic stress, and hormone signalling pathways (Vidal et al. 2014; da Silva et al. 2024). Despite its indispensable role, N availability often limits plant productivity in agricultural systems, where insufficient soil N directly constrains biomass accumulation and reduces harvestable yield, ultimately impacting grower profitability (Navarro-Morillo et al. 2024; Lopez et al. 2023). To counteract this limitation, farmers frequently apply substantial quantities of N fertilizers. However, crop uptake efficiency remains relatively low, with less than 50% of applied N being utilized effectively, while the remainder is lost through excessive rainfall or irrigation-driven nitrate leaching. These losses contribute to a fluctuating soil N supply, creating alternating periods of deficiency, sufficiency, or surplus. Such variability, whether driven by environmental conditions or agricultural practices, imposes significant metabolic stress on plants, disrupting key physiological functions and ultimately impairing growth and productivity (Dechorgnat et al. 2019). Addressing this challenge necessitates optimized N management strategies that improve nutrient retention and enhance plant NUE to sustain crop yields while minimizing environmental impacts (Govindasamy et al. 2023).

Previous research has demonstrated that different static N levels and availability impact various plant processes. For instance, high N levels was associated with increased shoot growth and total biomass, enhanced photosynthesis, but inhibited root development in apple (Zhao et al. 2020). Conversely, low N treatment increased root biomass, promoting higher root-to-shoot (R/S) ratio, which facilitate effective carbon (C) assimilate allocation to the shoot as an adaptive strategy against N limited environments (Plett et al. 2016; Zhao et al. 2020; Lopez et al. 2023; Zhang et al. 2021; Sun et al. 2023). Optimal static N supply has also been shown to modulate growth, enhance photosynthesis and improve N and C assimilation, as well as increase sugar metabolism in different plants (Peng et al. 2023; George et al. 2016; Chen et al. 2004; Zhao et al. 2020). Although these studies provide comprehensive insight into the impacts of varying static N supply on growth and C dynamics in various plant species, they overlook the complexity of changing N availability encountered in real-world agricultural settings.

There remains a significant gap in understanding the physiological and biochemical responses of plants when N concentrations are supplemented or increased after a period of limitation. Considering that both under-application and over-application of N fertilizers have detrimental effects not only on plant growth and metabolic processes but also on the environment and human health (Nacry et al. 2013; Zhang et al. 2021; Zhao et al. 2020), it is imperative to explore alternative approaches that support plant development under fluctuating environmental conditions. In this context, we propose that supplementing or altering N levels during plant growth may offer greater benefits than maintaining plants exclusively on static N supply. While static N supply has been extensively studied and documented in previous research (Sun et al. 2022; Nunes-Nesi et al. 2010; Paul and Stitt 1993; Zhang et al. 2021; Zhao et al. 2020), focusing on dynamic conditions, such as supplementing N after a period of limitation, provides critical insights for developing crops with higher yields and improved NUE, addressing global food security challenges. This study uniquely focuses on investigating the adaptive mechanisms by which plants respond to Supplemental Nitrogen (SN). We hypothesize that plants treated with SN leverage the increased N availability during the transition phase from a period of limited N supply as an adaptive mechanism. This adaptive response is expected to improve resource allocation and utilization, thereby promoting growth and enhancing photosynthesis.

This study specifically investigates the mechanisms underlying altered C allocation between the shoot and root in maize under SN conditions. To provide a comprehensive understanding of maize responses to dynamic N supply, we examined the effects of SN on growth, photosynthesis, and sugar and starch metabolism in both maize leaves and roots. Furthermore, we delved into the molecular signatures driving sugar metabolism by analysing the expression patterns of sucrose- and starch-metabolism-related genes in these tissues under SN treatment. We also conducted a detailed analysis of the diurnal and spatial sucrose and starch patterns under SN treatment conditions. The findings of this study provide a comprehensive understanding of SN-modulated carbon allocation and accumulation in maize, offering novel and valuable insights for optimizing crop production under the more realistic scenario of fluctuating N supply conditions.

## Materials and method

### Plant materials and experimental site

Seeds of the fast-flowering, short-cycle inbred mini-maize line TX-40 J (McCaw et al. 2016) were used in this study. This maize line offers significant advantages, including a uniform genetic background, a shorter lifecycle, and early flowering, which enable efficient and targeted research in various areas of plant biology. The traits observed in this variety serve as valuable references for breeders, facilitating the development of hybrids with enhanced performance and improved nitrogen use efficiency (McCaw et al. 2021). The seeds were germinated in Oasis Horticube Propagation Slabs (Aqua Gardening, Brisbane, Australia) placed in germination trays. The trays were transferred to a climate-controlled growth room, set to a 14/10 day-night cycle, with temperatures of 25 °C during the day and 22 °C at night, and 80% relative humidity for 5 d to allow for seed germination. Once the seedlings had germinated uniformly, they were transferred to a temperature controlled glasshouse for the subsequent experiments (Amoah and Kaiser 2025).

### Experimental treatment, set up and sampling

**Fig. 1:**
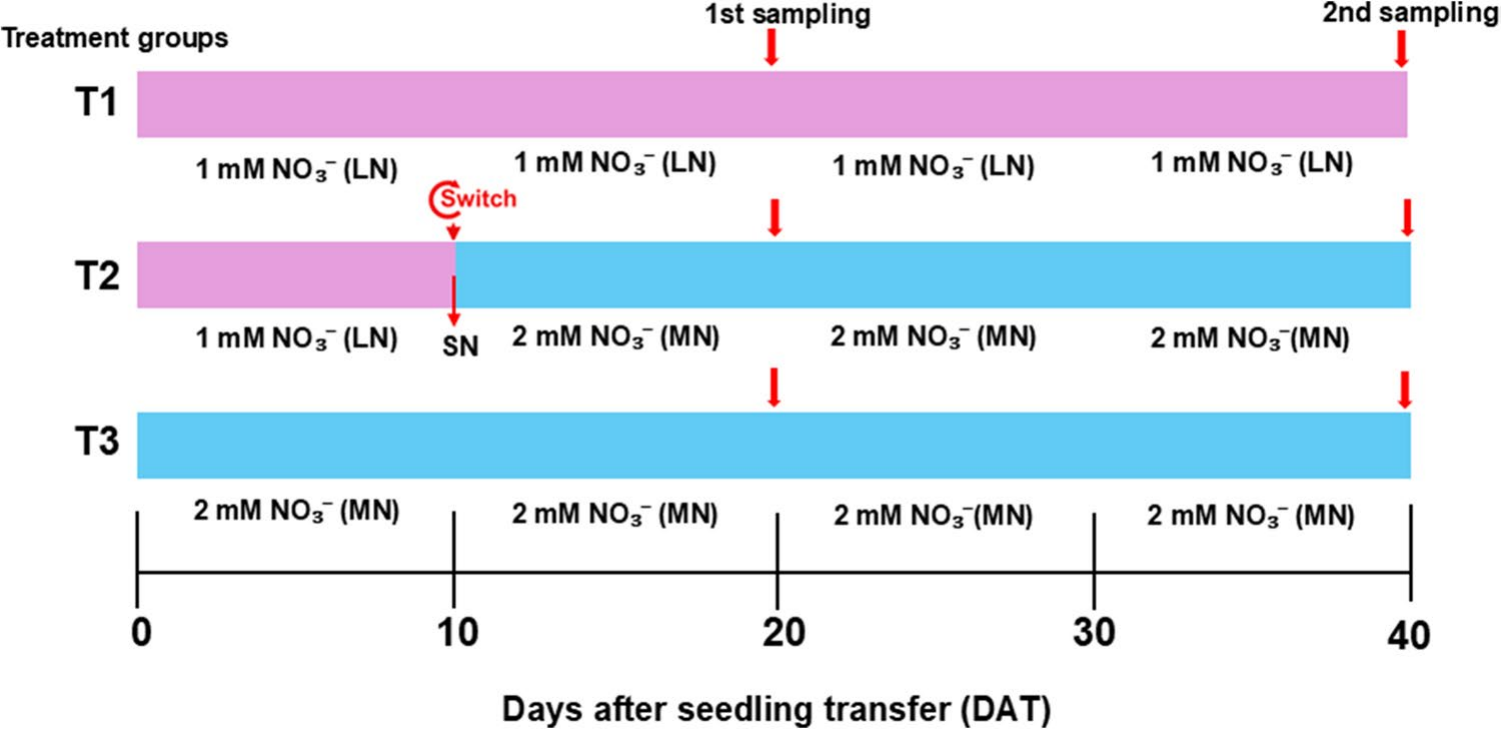
Schematic representation of the experimental setup for the maize inbred line TX-40 J. Maize seedlings were assigned to three nitrogen treatment groups (T1 to T3): T1, 1 mM NO₃⁻ (low N, LN); T2, supplementation of 1 mM NO₃⁻ with 2 mM NO₃⁻ at 10 days after transfer (DAT) (supplemental N, SN); and T3, 2 mM NO₃⁻ (medium N, MN). Plants were subsequently grown for an additional 30 days (up to 40 DAT), with shoot, root, and leaf samples collected at 20 DAT and 40 DAT for independent plant replicates. In the diagram, orange and green blocks represent 1 mM NO₃⁻ and 1 mM NO₃⁻. Red downward arrows indicate the nitrogen level supplementation (SN) and tissue sampling time points

Uniformly grown seedlings were divided into three treatment groups (T1–T3) and cultivated in 3 L pots, with their roots supported by inorganic expanded clay pellets (Aqua Gardening, Brisbane, Australia). Upon reaching the fully expanded third leaf stage (Fig. 1) (Amoah and Kaiser 2025), plants in T1 and T2 were initially supplied with 1 mM NO₃⁻, while T3 received 2 mM NO₃⁻. T1 and T3 plants continued growing on their respective static N levels, 1 mM NO₃⁻ (low nitrogen, LN) and 2 mM NO₃⁻ (moderate nitrogen, MN). In contrast, T2 plants underwent N supplementation (SN), where the initial 1 mM NO₃⁻ was replaced with 2 mM NO₃⁻ (1 mM NO₃⁻ → 2 mM NO₃⁻). Thus, T2 served as the primary treatment for assessing the effects of SN on C metabolism in maize, while T1 and T3 functioned as control groups. Low NO₃⁻ concentrations were deliberately used to better reflect field conditions and enable a clearer evaluation of nitrogen assimilation dynamics. This approach facilitates the assessment of NUE, reveals key physiological and molecular adaptations, and allows precise analysis of NO₃⁻ and NH₄⁺ interactions (Ye et al. 2022; Amoah et al. 2025). The plants were grown for 40 days after seedling transfer (DAT), with sampling performed for the shoot, leaf and roots at 20 and 40 DAT (Amoah and Kaiser 2025). The system was set up in a climate-controlled glasshouse, with conditions matching those of the growth room used for seed germination, but with supplemental LED lighting providing 1000 µmol m⁻^2^ s⁻^1^ at pot level. Each system was designed to accommodate 40 pots, with one plant per pot. Plants were drip-irrigated with the respective nutrient solution, which was circulated through a hydroponic pump system. Irrigation occurred twice daily for 1 min, at 12:00 PM and 5:00 PM (Amoah and Kaiser 2025).

The nutrient solution comprised the following concentrations (in mM): 1.0 or 2.0 KNO_3_, 1.0 1.0 MgSO₄, 1.0 KH₂PO₄, 0.05 H₃BO₃, 0.005 MnSO₄, 0.001 ZnSO₄, 0.001 CuSO₄, 0.001 Na₂MoO₄, 0.1 KCl, 0.1 Fe-EDTA, 0.1 Fe-EDDHA, 2.5 Ca(NO₃)₂, 0.25 K₂SO₄, 0.25 CaCl₂, and 1.75 CaSO₄ (Amoah and Kaiser 2025) and were stored in 162 L Brute Containers with lids (Rubbermaid, USA). The solution was changed weekly, with daily pH adjustments with 1 M H_2_SO_4_ or 1 M NaOH to maintain a stable pH of 5.9. The treatment solution was delivered to the system by an Eden 140G FL submersible water pump (Creative Pumps, Australia). Plants were uniquely identified and randomized into blocks using *agricolae* package of R statistical software (v4.4.5). Fresh leaf and root tissues for biochemical analysis were collected, immediately frozen in liquid nitrogen (N₂), and stored at −80 °C. Shoot and root samples for biomass analysis were oven-dried at 70 °C for 48 h to determine dry weights (DW). The shoot and root biomass values were summed to calculate the total plant biomass in grams (g). All analyses were conducted using distinct, independent plant replicates to ensure statistical robustness and avoid repeated sampling from the same individual.

### Spatial distribution and diurnal changes determination

To examine the spatial distribution of sucrose and starch, the youngest emerging leaf of independent plant replicates at 20 and 40 DAT was sampled and divided into upper and middle sections. Additional samples included the corresponding leaf sheath, root and developing ear. Sampling was done at 22:00 and again at 7:00 the following morning on 40 DAT. For diurnal analysis, the middle sections of the youngest fully expanded leaves were collected at 7:00, 12:00, 17:00, 22:00, and 7:00 (the next day) on 20 and 40 DAT. All samples were immediately frozen in liquid N_2_ and stored at −80 °C for subsequent biochemical analysis.

### Net photosynthetic rate and chlorophyll and nitrogen (N) measurement

The net photosynthetic rate (Pn) was measured on the young emerging leaf of each treatment using the portable LI-6400 photosynthetic system (LI-COR Inc., Lincoln, NE, USA). Measurements were taken at 9:00 AM and 11:00 AM. Cuvette conditions included a light level of 1000 µmol m⁻^2^ s⁻^1^, CO₂ concentration of 400 ppm, flow rate of 500 µmol m⁻^2^ s⁻^1^, and relative humidity between 60 and 65%. Chlorophyll pigment was extracted from approximately 0.1 g of leaf tissue using 100% methanol on a shaker at 25 °C until the tissue was completely bleached. The extract was then centrifuged at 10,000 × *g* for 10 min, and the absorbance of the supernatant was measured at 646, 470, and 663 nm using a Uv–vis spectrophotometer (Shimadzu, Tokyo, Japan). The concentration of chlorophyll was calculated following the method described by Amoah and Kaiser (2025).

N content was determined using the Kjeldahl method as described by Amoah and Kaiser (2025), with minor modifications. A 0.2 g dry sample was digested with 0.5 mL of concentrated H₂SO₄ and 0.5 mL of a catalyst mixture consisting of 10 g of K₂SO₄ and 1 g of CuSO₄. The mixture was heated at 100 °C for 60 min on a heating block in a fume hood. After digestion, the samples were allowed to cool, and 0.5 mL of 40% NaOH solution was slowly added, followed by 0.5 mL of distilled water. Subsequently, 1 mL of the resulting mixture was combined with 1 mL of Nessler’s reagent and incubated for 10 min at room temperature. The absorbance was measured at 420 nm using a UV–Vis spectrophotometer (Shimadzu, Tokyo, Japan). N content was determined from a standard curve generated with (NH₄)₂SO₄ standards.

### Total protein and amino acid protein quantification

Total amino acids content was determined using the methods outlined by (Bates et al. 1973). Frozen leaf tissues (100 mg) were homogenized in 10 mL of 3% (v/v) aqueous sulfosalicylic acid. After filtering the homogenate, 1 mL of filtrate was combined with 1 mL of glacial acetic acid and 1 mL of acidic ninhydrin. The resulting mixture was incubated at 100 °C for 1 h and then cooled on ice for 20 min before being extracted with 1 mL of toluene. The concentrations of amino acids were measured using a spectrophotometer at A580 nm. Leaf and root total protein contents were extracted from 100 mg of fresh material in 1 mL chilled extraction buffer [50 mM HEPES pH 7.5, 20% glycerol, 1 mM EDTA, 1 mM EGTA, 0.1% Triton X-100, 1 mM Benzamidine, 1 mM 6-Aminohexanoic acid and 1% Protease Inhibitor Cocktail (Sigma-Aldrich cat#P9599)] and assayed following manufacturer’s instruction.

### Soluble sugar, starch, glucose and sucrose content determination

Soluble sugar and sucrose content were measured as described by Amoah and Kaiser (2025). Briefly, 100 mg of ground samples were homogenized in 1 mL of 80% (v/v) ethanol, and the mixture was heated at 80 °C for 30 min. After cooling for 5 min, the mixture was centrifuged at 12,000 × *g* for 10 min. The supernatants were collected, and soluble sugar and sucrose contents were determined by measuring absorbance at 620 nm and 480 nm, respectively, using a Uv–Vis spectrophotometer (Shimadzu, Tokyo, Japan). The ethanol-insoluble residue was used for starch extraction following the procedure outlined by Amoah et al. (2024). After evaporating the ethanol, 2 mL of distilled water was added to the samples, which were then incubated at 100 °C for 15 min. Starch was hydrolyzed using separate treatments of 9.2 M and 4.6 M HClO₄. The starch content was quantified spectrophotometrically using the anthrone reagent, and absorbance was measured at 620 nm.

Glucose and fructose content were determined using the anthrone colorimetry method as described by Dong et al. (2023). A mixture of 1 mL of supernatant and 5 mL of anthrone diluted sulfuric acid reagent was boiled for 10 min. A blank was prepared similarly, using 1 mL of distilled Milli-Q water instead of the supernatant. After cooling, the solution's absorbance was measured at 620 nm using a spectrophotometer, with the blank adjusted to zero. For fructose content, 1 mL of extract, 1 mL of 0.1% (v/v) hydroquinone, and 3.5 mL of 30% (v/v) HCl were combined in a test tube, thoroughly mixed, and heated at 80 °C for 10 min in a water bath. After cooling, the solution's absorbance was measured at 480 nm using a s Uv–Vis spectrophotometer (Shimadzu, Tokyo, Japan), with the blank adjusted to zero. The measured absorbance was used to calculate fructose content based on a standard curve.

### Sugar metabolism enzymes activity assays

Proteins containing enzymatic activity was determined following the method described by Chen et al. (2019), with slight modifications. A 100 mg plant tissue sample was ground to a fine powder using liquid N_2_ and homogenized in 1 mL of ice-cold extraction buffer. The buffer contained 50 mM HEPES–NaOH (pH 7.5), 5 mM MgCl₂, 0.1% (v/v) β-mercaptoethanol, 0.05% (v/v) Triton-X100, 0.05% (w/v) BSA, 2% (w/v) polyvinylpyrrolidone, and 1 mM EDTA. The homogenate was centrifuged at 12,000 × *g* for 10 min at 4 °C, and the supernatant was used for the assays of sucrose synthase, vacuolar invertase, and cytoplasmic invertase (CINV) activities. The pellet was washed twice with 0.5 mL of extraction buffer and then resuspended in 1.8 mL of salt extraction buffer. Samples were extracted overnight at 4 °C and centrifuged at 12 000 × *g* for 10 min at 4 °C. The resulting supernatant was used for the assay of cell wall invertase activity.

Sucrose synthase and cytoplasmic invertase activities were determined following the method by Li et al. (2021), while vacuolar invertase activity was assayed according to Chen et al. (2019). For sucrose synthase activity, 100 μL of enzyme extract was mixed on ice with 200 μL of a reaction solution containing 80 mM MES-NaOH (pH 5.5), 5 mM NaF, 100 mM sucrose, and 5 mM UDP. The reaction was incubated at 30 °C for 30 min and terminated by boiling for 2 min. A control reaction lacking UDP was included. For vacuolar, cell wall and cytoplasmic invertase assays, the procedure was similar, but with variations in the reaction mixtures. Vacuolar invertase activity was measured using 200 mM acetic acid-sodium acetate buffer (pH 4.5 or 4.8) with 100 mM sucrose. Cytoplasmic invertase activity utilized 100 mM HEPES–NaOH buffer (pH 7.5) with 100 mM sucrose. Sucrose phosphate synthase (SPS) activity, 0.1 g of frozen tissue was homogenized in an extraction buffer containing 50 mM Tris–HCl (pH 7.5), 1 mM EDTA, 1 mM MgCl_2_, 12.5% (v/v) glycerine, 10% polyvinylpyrrolidone (PVP), and 10 mM mercaptoethanol. For sucrose phosphate synthase (SPS) activity, 200 μL of supernatant was mixed with reaction buffer containing 200 mM Tris–HCl (pH 7.0), 40 mM MgCl_2_, 12 mM UDPglucose, 40 mM fructose-6-P, and 200 μL extract. Another reaction buffer containing 12 mM UDP, 40 mM sucrose, 200 mM Tris–HCl (pH 7.0), and 40 mM MgCl_2_ was prepared (Liu et al. 2013). The mixture was incubated at 30 °C for 30 min and terminated using 100 μL 2 mol L^−1^ of NaOH. The mixture was then heated at 100 °C for 10 min to destroy untreated hexose and hexose phosphates, cooled to room temperature, and mixed with 1 mL of 0.1% (w/v) resorcin in 95% (v/v) ethanol and 3.5 mL of 30% (w/v) HCl. The solution was incubated for 10 min at 80 °C. Sucrose content in the SPS reaction was calculated using a standard curve measured at A480 nm.

### Starch metabolism enzymes activity assay

For starch synthase (SS) activity, 100 mg of tissue samples were homogenized in an extraction buffer containing 50 mM Tris–HCl (pH 7.0), 10% glycerol, 10 mM EDTA, 5 mM DTT, 1 mM PMSF, and 50 μL/g tissue of 10 × Protease Inhibitor Cocktail (Sigma-Aldrich, Cat# P9599) (Cao et al. 1999). The homogenate was centrifuged at 12,000 × g for 10 min, and the supernatant was collected. A reaction mixture was prepared by mixing 0.1 mL of the supernatant with 0.9 mL of a solution containing 50 mM Tris–HCl (pH 7.0), 5 mM ADP-glucose, 1 mg/mL glycogen, and 10 mM MgCl₂. The reaction mixture was incubated at 30 °C for 30 min. To stop the reaction, 0.1 M HCl was added to denature the enzymes. To detect inorganic phosphate (Pi) consumption, 1% (w/v) ammonium molybdate was added. The mixture was incubated at room temperature for 30 min, and the absorbance was recorded at a wavelength of 620 nm using a UV–Vis spectrophotometer (Shimadzu, Tokyo, Japan). A standard curve was prepared using known Pi concentrations, and starch synthase activity was calculated as the amount of Pi released, expressed in μmolg⁻^1^FW.

The activities of α-and β-amylase was measured according to the methods by Liu et al. (2013) with minor modifications. Briefly, tissue samples were homogenized in 1 mL of chilled distilled water and centrifuged at 12,000 × *g* for 15 min. The supernatants were separated and used for quantifying α-and β-amylase. For α-amylase activity, 0.5 mL of supernatant was mixed with 3 mM CaCl_2_, heated at 70 °C for 5 min to inactivate β-amylase, cooled to room temperature, followed by the addition of 2% starch solution in 0.1 M citrate buffer. The mixture was incubated at 30 °C for 5 min and stopped by adding 1 mL of colour reagent (dinitro salicylic acid). The mixture was heated at 50 °C for 5 min, cooled down, and the α-amylase activity was determined by recording absorbance at 540 nm wavelength with a Uv–Vis spectrophotometer (Shimadzu, Tokyo, Japan). β-amylase activity was assayed by initially inactivating α-amylase with 0.1 M EDTA. After, a 1 mL solution containing 0.1 M EDTA, 2% starch solution, and 0.1 mM citrate buffer were mixed with 0.5 mL enzyme extract. The mixture was incubated at 30 °C for 5 min. The reaction was stopped by adding 1 mL of colour reagent (dinitro salicylic acid). The β-amylase activity was measured by recording absorbance at 540 nm wavelength with a Uv–vis spectrophotometer (Shimadzu, Tokyo, Japan).

ADP-glucose pyrophosphorylase (AGPase) activity was determined using previously described methods by Ali et al. (2019), with minor modifications. Briefly, 100 mg of fresh samples were homogenized in 1 mL of ice-cold extraction buffer containing 0.1 M Tris–HCl (pH 7.9), 5 mM glutathione, and 1 mM EDTA. The homogenate was centrifuged at 15,000 × *g* for 20 min at 4 °C, and the supernatant was collected. Subsequently, 0.1 mL of the supernatant was mixed with 0.9 mL of a reaction mixture containing 0.4 M Tris–HCl buffer (pH 7.9), 0.06 M MgSO₄, 48 mM cysteine, 2.4 mg/mL BSA, 4 mM ADP-glucose, 20 mM sodium pyrophosphate, 30 mM 3-phosphoglycerate, and 4 units each of glucose-6-phosphate dehydrogenase and phosphoglucomutase. Afterwards, 0.1 mL of enzyme extract was added to NADP⁺ as the final component. The absorbance was measured at 340 nm using a UV–Vis spectrophotometer (Shimadzu, Tokyo, Japan). The AGPase activity was expressed as μmol min⁻^1^ g⁻^1^ FW.

### RNA isolation, cDNA synthesis and qPCR analysis

Total RNA was isolated from leaf and root tissues of independent plant replicates using the TRIzol RNA Isolation Reagents (Invitrogen, Carlsbad, CA, USA) following the manufacturer’s protocol. RNA quantity and integrity were assessed by measuring the optical density at 260 nm and through 1.0% (w/v) agarose gel electrophoresis, respectively. Subsequently, 1 µg of total RNA was reverse-transcribed into single-stranded cDNA using the iScript™ RT Reagent Kit (Bio-Rad, Hercules, CA, USA) according to the manufacturer’s instructions. Quantitative real-time polymerase chain reaction (qPCR) was performed using the CFX 96 Real-Time System (Bio-Rad, Richmond, CA, USA) with SYBR Green fluorescence (Bio-Rad, Richmond, CA, USA). The ^∆∆^CT method was used for data analysis. Gene-specific primers used in our previous experiment (Table S1) was applied to assess their expression patterns under different N (LN, MN, and HN) treatment conditions. The thermal cycling conditions consisted of an initial denaturation step at 95 °C for 5 min, followed by 40 cycles of 95 °C for 15 s, 55 °C for 15 s, and 72 °C for 30 s. All experiments were conducted with three biological replicates, and relative transcript levels were normalized using *ZmActin1* and *ZmUBQ1* as internal controls.

### Statistical analysis

The experiment was conducted twice, and tissue samples were collected in biological triplicate. Data were analysed using two-way ANOVA followed by Tukey’s HSD post hoc test (GraphPad Prism, version 10). Values represent the mean ± SE of six independent plants (*n* = 6). Statistical significance was denoted as *P* ≤ 0.05 (*), *P* ≤ 0.01 (**), *P* ≤ 0.001 (***), *P* ≤ 0.0001 (****), and not significant (ns). Graphs were generated in GraphPad Prism (v10.4.0), and Pearson’s correlation analyses were performed using the *ggcorrplot* package in R software R (v4.4.5).

## Results

### Effect of N availability on phenotypes, growth and photosynthesis activity

**Fig. 2:**
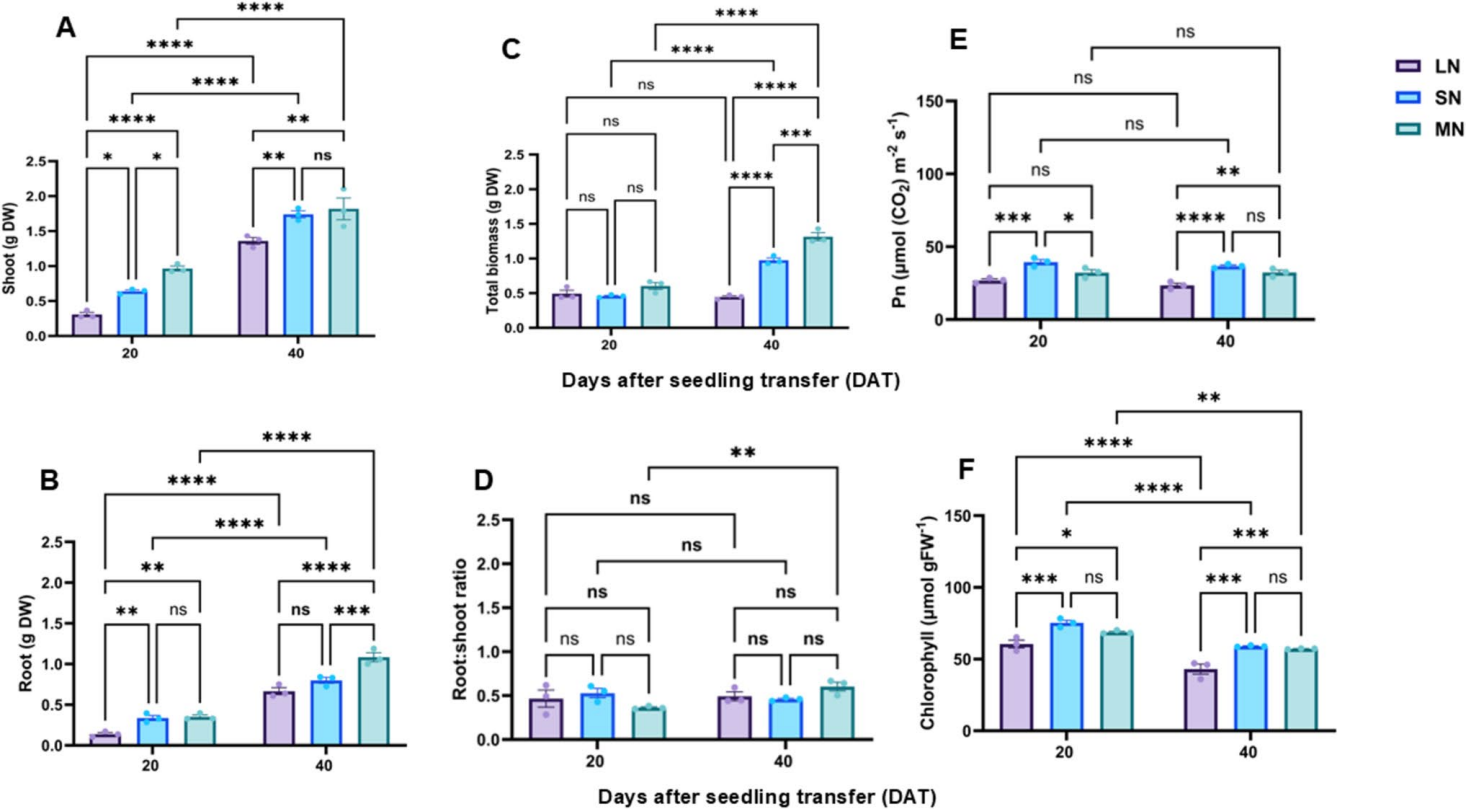
Shoot biomass (**A**), root biomass (**B**), total biomass (**C**), root: shoot ratio (**D**), photosynthetic rate (**E**) and leaf chlorophyll content under different nitrogen levels. Data points represent the mean ± standard error (SE) of six independent biological replicates (*n* = 6). *, **, ***, ****, and ns denote significance at *P* ≤ 0.05, 0.01, 0.001, 0.0001, and not significant, respectively. Statistical significance was determined using two-way analysis of variance (ANOVA), followed by Tukey’s HSD post-hoc test. DW, dry weight; FW, fresh weight; LN, low nitrogen (1 mM NO₃⁻); MN, medium nitrogen (2 mM NO₃⁻); SN, nitrogen level supplementation (1 mM NO₃⁻ → 1 mM NO₃⁻)

Maize seedlings exhibited distinct phenotypic responses to varying N levels. By 40 DAT, plants grown under SN treatment demonstrated enhanced shoot and root growth, resulting in superior overall performance compared to those under LN and MN treatments (Fig. S1A–B). Specifically, SN-treated plants developed vigorous shoots and roots and produced larger cobs. In contrast, plants subjected to other N treatments, particularly LN, displayed restricted shoot and root growth along with smaller cobs, underscoring the pronounced influence of N levels and availability on maize growth dynamics. Consistent with these phenotypic changes, shoot and root biomass varied significantly (*P* ≤ 0.05) among N treatments across different growth stages. At 20 DAT, MN-treated plants exhibited significantly increased shoot and total biomass, while SN-treated plants showed elevated root biomass (Fig. 2A–C). The R/S ratio did not differ significantly among treatments (Fig. 2D). However, at 40 DAT, MN and SN plants displayed modest, non-significant increases in shoot biomass, whereas root and total biomass were significantly higher in MN plants (Fig. 2A–D). Photosynthetic activity was higher across all treatments at 20 DAT but declined by 40 DAT. Comparatively, SN-treated plants maintained significantly elevated leaf Pn and chlorophyll content, whereas these indicators remained consistently and significantly lower under LN treatment (Fig. 2E–F).

### N availability differentially impacted total N, amino acid and protein contents

**Fig. 3:**
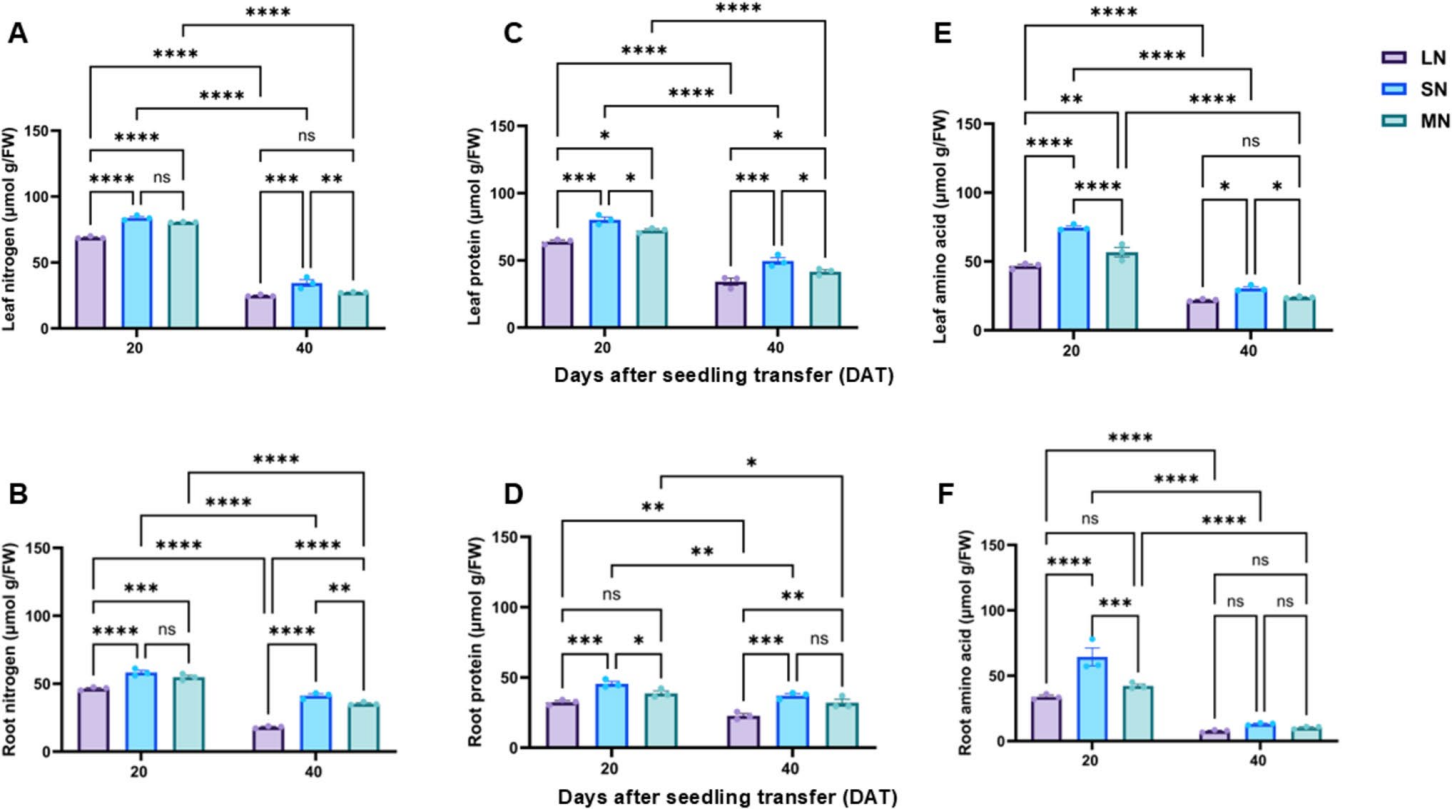
Leaf total nitrogen content (**A**), root total nitrogen content (**B**), leaf protein content (**C**), root protein content (**D**), leaf total amino acid content (**E**) and root total amino acid content under different nitrogen levels. Data points represent the mean ± standard error (SE) of six independent biological replicates (*n* = 6). *, **, ***, ****, and ns denote significance at *P* ≤ 0.05, 0.01, 0.001, 0.0001, and not significant, respectively. Statistical significance was determined using two-way analysis of variance (ANOVA), followed by Tukey’s HSD post-hoc test. FW, fresh weight; LN, low nitrogen (1 mM NO₃⁻); MN, medium nitrogen (2 mM NO₃⁻); SN, nitrogen level supplementation (1 mM NO₃⁻ → 1 mM NO₃⁻)

The N, total protein, and amino acid contents varied among the N treatment plants. These indicators were elevated at 20 DAT but declined significantly (*P* ≤ 0.05) by 40 DAT (Fig. 3A–F). SN plants exhibited the highest leaf and root N contents at both 20 and 40 DAT, with significant (*P* ≤ 0.05) differences compared to LN plants in both tissues at both time points and compared to MN plants in leaves and roots at 40 DAT only (Fig. 3A–B). Similarly, SN plants had the highest leaf and root protein and amino acid contents at both time points. Leaf total protein and amino acid levels differed significantly (*P* ≤ 0.05) among N treatments at both 20 and 40 DAT (Fig. 3C, E). In roots, protein and amino acid contents were significantly (*P* ≤ 0.05) different across all treatments at 20 DAT; however, at 40 DAT, differences (*P* ≤ 0.05) were observed only between LN and SN plants (Fig. 3E–F).

### Different N levels influenced the accumulation of sugars and starch, as well as the activity of key metabolizing enzymes

**Fig. 4:**
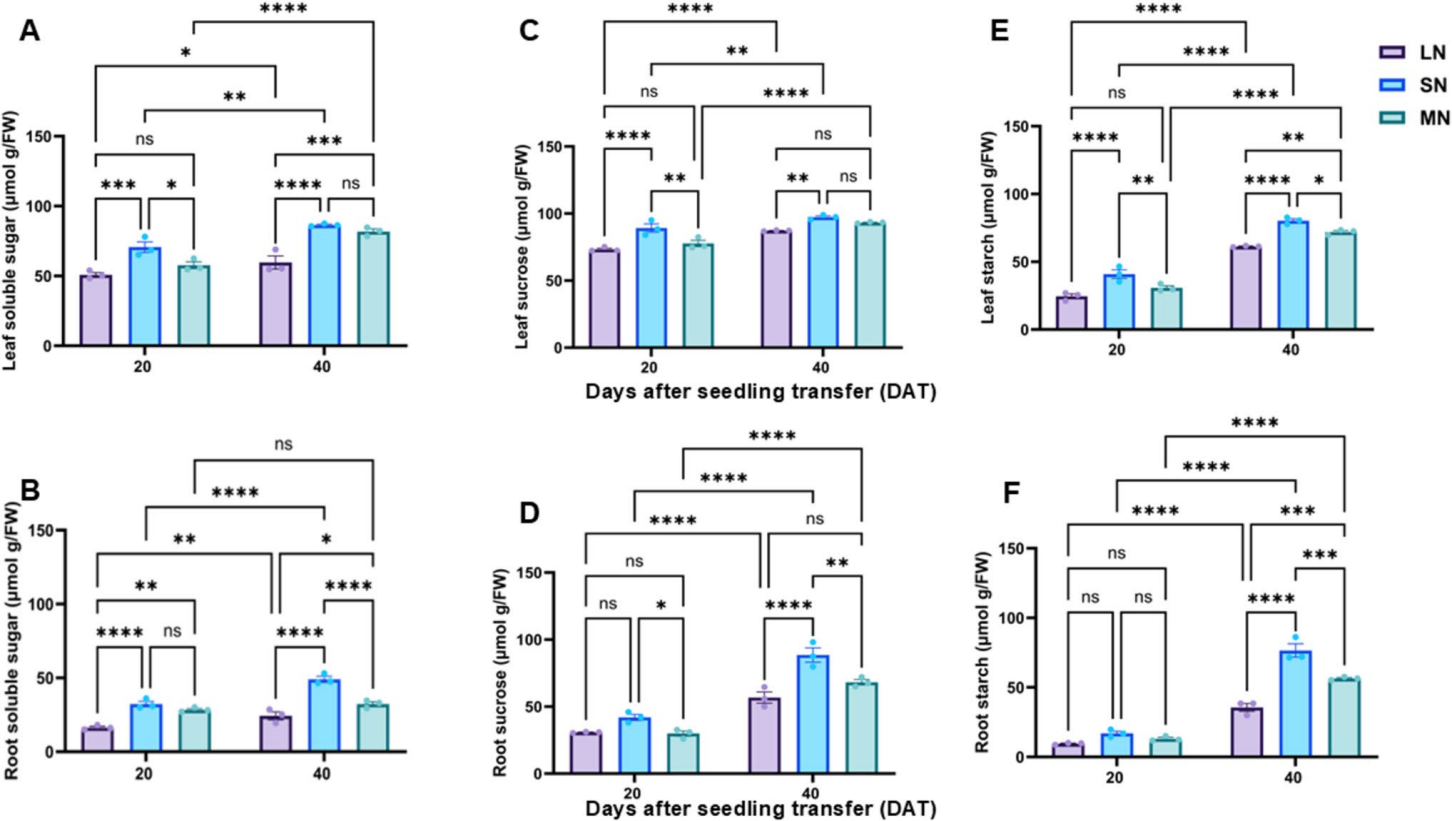
Leaf soluble sugar content (**A**), root soluble sugar content (**B**), leaf sucrose content (**C**), root sucrose content (**D**), leaf starch content (**E**) and root starch content under different nitrogen levels. Data points represent the mean ± standard error (SE) of six independent biological replicates (*n* = 6). *, **, ***, ****, and ns denote significance at *P* ≤ 0.05, 0.01, 0.001, 0.0001, and not significant, respectively. Statistical significance was determined using two-way analysis of variance (ANOVA), followed by Tukey’s HSD post-hoc test. FW, fresh weight; LN, low nitrogen (1 mM NO₃⁻); MN, medium nitrogen (2 mM NO₃⁻); SN, nitrogen level supplementation (1 mM NO₃⁻ → 1 mM NO₃⁻)

Fructose and glucose accumulated differentially in the leaves and roots of maize seedlings under varying N treatments. Levels of both sugars were elevated at 20 DAT but declined significantly (*P* ≤ 0.05) by 40 DAT (Fig. S2A–D). At 20 DAT, fructose content differed significantly (*P* ≤ 0.05) among all treatments in both leaves and roots (Fig. S2A–B), whereas glucose accumulation showed significant (*P* ≤ 0.05) differences only in the roots (Fig. S2C–D). In contrast, soluble sugars, sucrose, and starch were lower at 20 DAT but increased substantially (*P* ≤ 0.05) by 40 DAT, with SN plants exhibiting a higher rate of accumulation compared to LN and MN treatments (Fig. 4A–F). At 20 DAT, significant (*P* ≤ 0.05) differences in soluble sugar and sucrose levels were observed in the leaves across all treatments. However, apart from starch, which showed no significant (*P* ≤ 0.05) variation in root tissues, the soluble sugar and sucrose contents differed significantly (*P* ≤ 0.05) among N treatments (Fig. 4C–D). By 40 DAT, the accumulation of soluble sugars, sucrose, and starch differed significantly (*P* ≤ 0.05) in both leaves and roots across all N treatments (Fig. 4A–F).

**Fig. 5:**
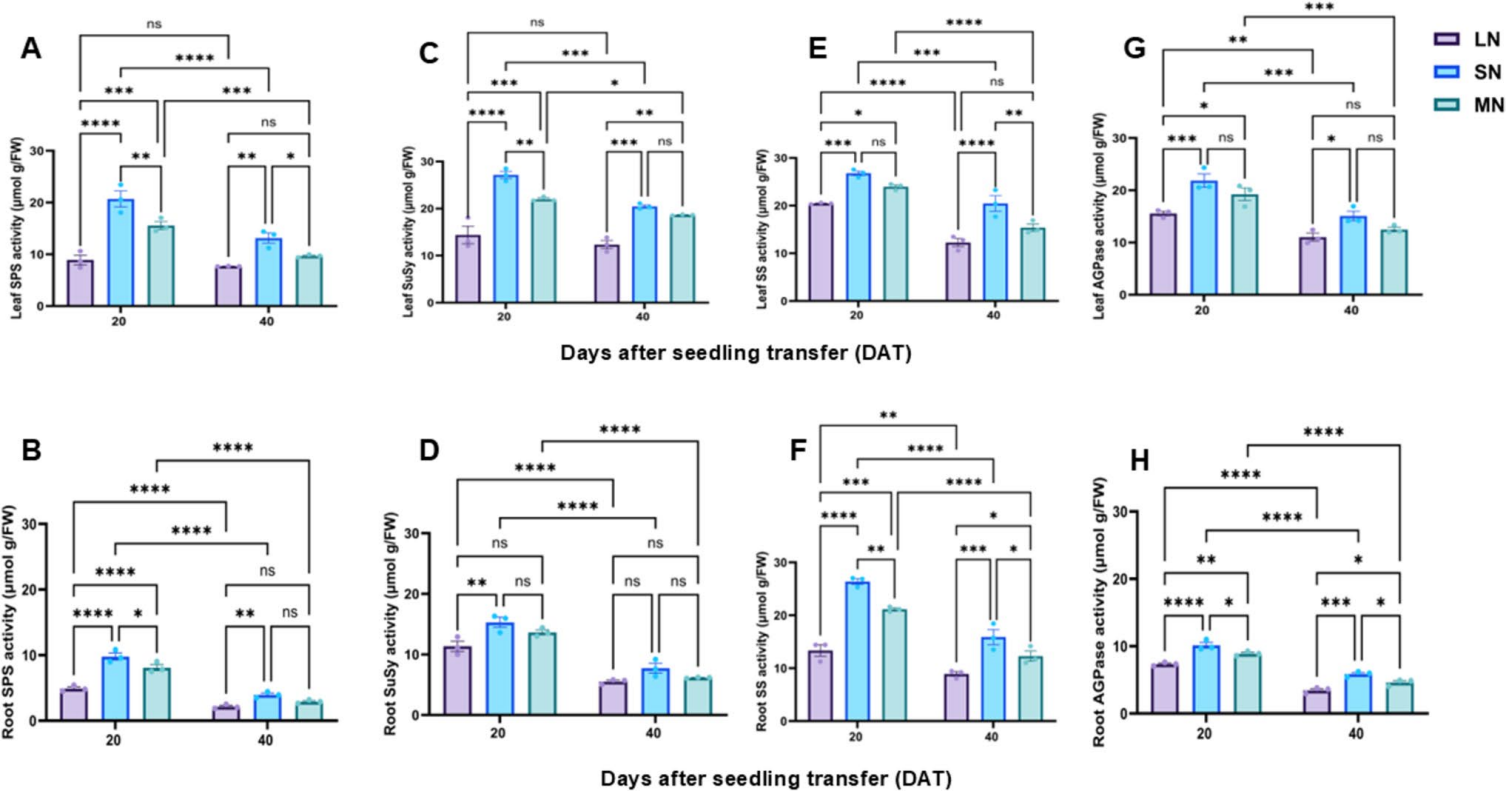
Leaf sucrose phosphate synthase activity (**A**), root sucrose phosphate synthase activity (**B**), leaf sucrose synthase activity (**C**), root synthase activity (**D**), leaf starch synthase activity (**E**) and root starch synthase activity, leaf ADP-glucose pyrophosphorylase activity (**F**) and root ADP-glucose pyrophosphorylase activity (**H**) under different nitrogen levels. Data points represent the mean ± standard error (SE) of six independent biological replicates (*n* = 6). *, **, ***, ****, and ns denote significance at *P* ≤ 0.05, 0.01, 0.001, 0.0001, and not significant, respectively. Statistical significance was determined using two-way analysis of variance (ANOVA), followed by Tukey’s HSD post-hoc test. FW, fresh weight; LN, low nitrogen (1 mM NO₃⁻); MN, medium nitrogen (2 mM NO₃⁻); SN, nitrogen level supplementation (1 mM NO₃⁻ → 1 mM NO₃⁻)

Consistent with soluble sugar and starch levels, the activities of sugar-metabolizing enzymes, SPS, SuSy, CINV, and VINV were differentially regulated in the leaves and roots of maize seedlings across N treatments and growth stages. Enzyme activities were elevated in both leaves and roots at 20 DAT but declined by 40 DAT, with SN-treated plants exhibiting a notable upregulation compared to LN and MN plants (Figs. 5A–D and S3A–D). With the exception of root SuSy activity, which showed no significant variation among N treatments at 40 DAT, SPS, CINV, and VINV activities in leaves and roots differed significantly across treatments at both time points. Similarly, starch-metabolizing enzymes, SS, AGPase, AMY, and BAM, showed higher activity at 20 DAT, followed by a decline at 40 DAT (Figs. 5E–F and S3E–F), with SN plants maintaining significantly (*P* ≤ 0.05) elevated enzyme activities in both leaves and roots. Whiles MN plants exhibited an optimum regulation of these enzymes in these tissues, LN treatment was associated with a relatively lower activities of all these enzymes (Figs. 5A-H, S3A-H).

### Sugars and starch metabolism-related genes were differentially regulated under different N levels

**Fig. 6:**
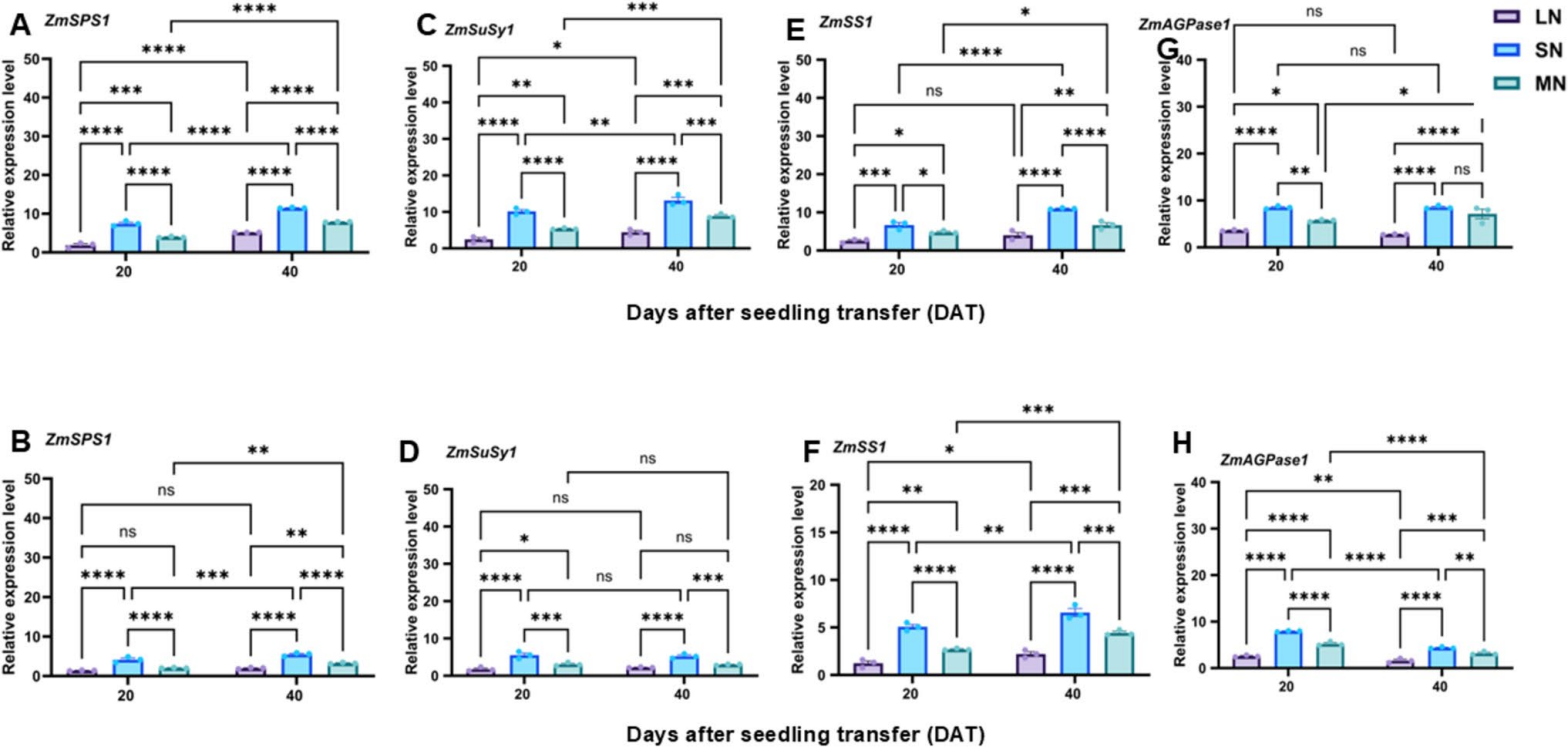
Expression patterns of sucrose and starch metabolism enzymes under different N levels. Relative expression levels of *ZmSPS1*, *ZmSuSy1*, *ZmSS1* and *ZmAGPase1* in the leaf (**A**, **C**, **E** and **G**) and *ZmSPS1*, *ZmSuSy1*, *ZmSS1* and *ZmAGPase1* in the root (**B**, **D**, **F** and **H**) of the maize inbred line TX-40 J. Data points represent the mean ± standard error (SE) of six independent biological replicates (*n* = 6). *, **, ***, ****, and ns denote significance at *P* ≤ 0.05, 0.01, 0.001, 0.0001, and not significant, respectively. Statistical significance was determined using two-way analysis of variance (ANOVA), followed by Tukey’s HSD post-hoc test. FW, fresh weight; LN, low nitrogen (1 mM NO₃⁻); MN, medium nitrogen (2 mM NO₃⁻); SN, nitrogen level supplementation (1 mM NO₃⁻ → 1 mM NO₃⁻)

**Fig. 7:**
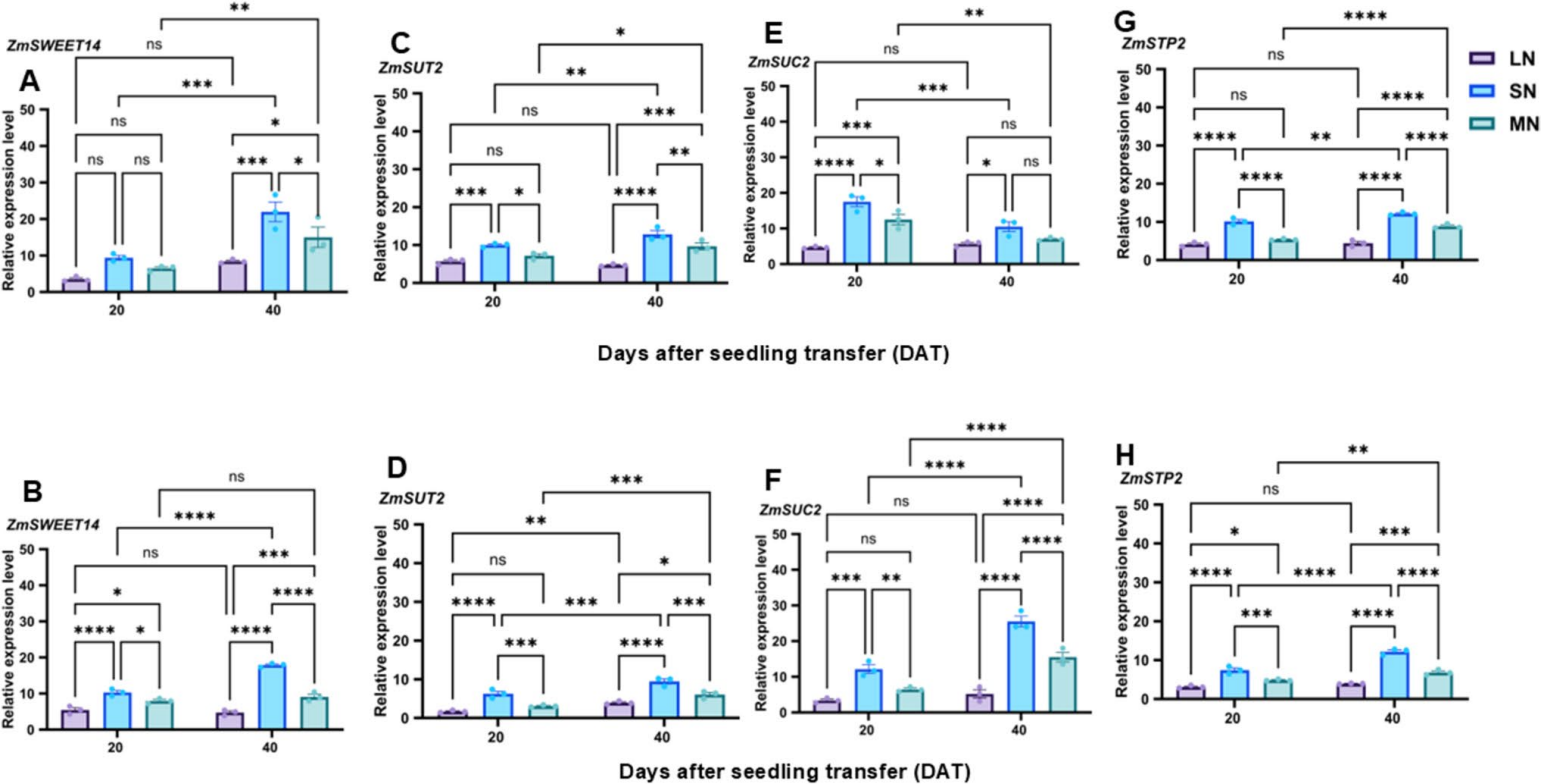
Expression patterns of sucrose transporter genes under different N levels. Relative expression levels of *ZmSWEET14*, *ZmSUT2*, *ZmSUC2* and *ZmSTP2* in the leaf (**A**, **C**, **E** and** G**) and *ZmSWEET14*, *ZmSUT2*, *ZmSUC2* and *ZmSTP2* in the root (**B**, **D**, **F** and **H**) of the maize inbred line TX-40 J. Data points represent the mean ± standard error (SE) of six independent biological replicates (*n* = 6). *, **, ***, ****, and ns denote significance at *P* ≤ 0.05, 0.01, 0.001, 0.0001, and not significant, respectively. Statistical significance was determined using two-way analysis of variance (ANOVA), followed by Tukey’s HSD post-hoc test. FW, fresh weight; LN, low nitrogen (1 mM NO₃⁻); MN, medium nitrogen (2 mM NO₃⁻); SN, nitrogen level supplementation (1 mM NO₃⁻ → 1 mM NO₃⁻)

The expression pattern of sugar metabolism (*ZmSPS1*, *ZmSuSy1*, *ZmSUT2*, *ZmSUC2*, *ZmSWEET14*, *ZmSTP2*, *ZmCINV1*, *ZmVINV1* and starch metabolism-related (*ZmSS1*, *ZmAGPase1*, *ZmAMY1* and *ZmBAM1*) were analysed in the leaves and roots of maize seedlings under LN, SN and MN treatment conditions. As seen in Fig. 6, the sugars and starch metabolism-related genes were differentially regulated under the different N levels. The sugar metabolism-related transcripts were upregulated in all treatment plants, and were relatively upregulated at 20 DAT than 40 DAT. However, the magnitude of expression of these genes were abundant in the leaves and roots of SN plant than LN and MN plants. For instance, in the leaves, the fold expression of these genes ranged from 0.8- (*ZmSUT2*) to 5.2-fold (*ZmSWEET14*) in LN plants, from 1.5-fold (*ZmSuSy1*) to 3.6-fold (*ZmSWEET14*) and from 1.2-fold (*ZmSUT2*) to 3.8-fold (*ZmSWEET14*) in MN plants. Similarly in the roots, the fold expression ranged from 1.2-fold (*ZmSPS1*) to 2.9-fold (*ZmSUC2*) in LN plants, from 1.3-fold (*ZmSuSy1*) to 7.8-fold (*ZmSUC2*) in SN plants and from 1.15-fold (*ZmSuSy1*) to 6.8-fold (*ZmSUC2*) in MN plants (Figs. 6A-D, 7A-H). Furthermore, the expression of starch metabolism-related genes, *ZmSS1*, *ZmAGPase1*, *ZmAMY1* and *ZmBAM1* were differentially upregulated in the leaves and roots of all treatment and was higher at 20 DAT than 40 DAT, with the higher expression pattern observed in plants under SN than LN or MN treatments. Specifically, the expression of these genes ranged from 1.2-fold (*ZmAMY1*) to 2.2-fold (*ZmAGPase1* in LN plants, from 2.2-fold (*ZmSS1*) to 3.4-fold (*ZmAMY1*) in SN plants and from 1.7-fold (*ZmSS1*) to 2.8-fold (*ZmAGPase1*) in the leaves of MN plants. In the root tissues, the fold expression of these genes ranged from 0.6-fold (*ZmAMY1*) to 2.3-fold (*ZmBAM1*), from 1.2-fold (*ZmAMY1*) to 2.6-fold (*ZmBAM*1) and from 1.3-fold (*ZmAGPase1*) to 2.0-fold (*ZmSS1*) in MN plants (Fig. 6E-H).

### N availability differentially enhanced diurnal sucrose and starch dynamics in leaves

Sucrose and starch accumulated in the leaves of all treatment plants throughout the day. However, the magnitude of sucrose and starch accumulation in the leaves was higher in SN plants compared with plants under LN and MN treatment. Whiles MN plants maintained an optimum sucrose and starch accumulation, LN plants showed lower sucrose and starch accumulation. As seen in Fig. 7A and C, the diel sucrose accumulation increased after 12:00, peaked at 17:00, and declined at 22:00, lasting until the following morning. Similarly, starch content was high in the morning, increased at 12:00, peaked at 17:00, and declined at 22:00, continuing until 7:00 the next day (Fig. 7B and D). Interestingly, these diel patterns for sucrose and starch remained consistent in all treatment plants at both 20 and 40 DAT. Specifically, at 20 DAT, significant differences (*P* ≤ 0.05) in sucrose and starch levels were observed among treatment groups, with SN-treated plants consistently exhibiting higher sucrose and starch levels throughout the day. These levels followed distinct daily rhythms: sucrose and starch concentrations increased after 12:00, declined at 22:00, and rose again by the next morning. After overnight transport, SN-treated plants retained significantly (*P* ≤ 0.05) higher sucrose and starch levels than LN and MN plants.

### Spatial distribution of sucrose and starch in the leaves of maize under different N levels

**Fig. 8:**
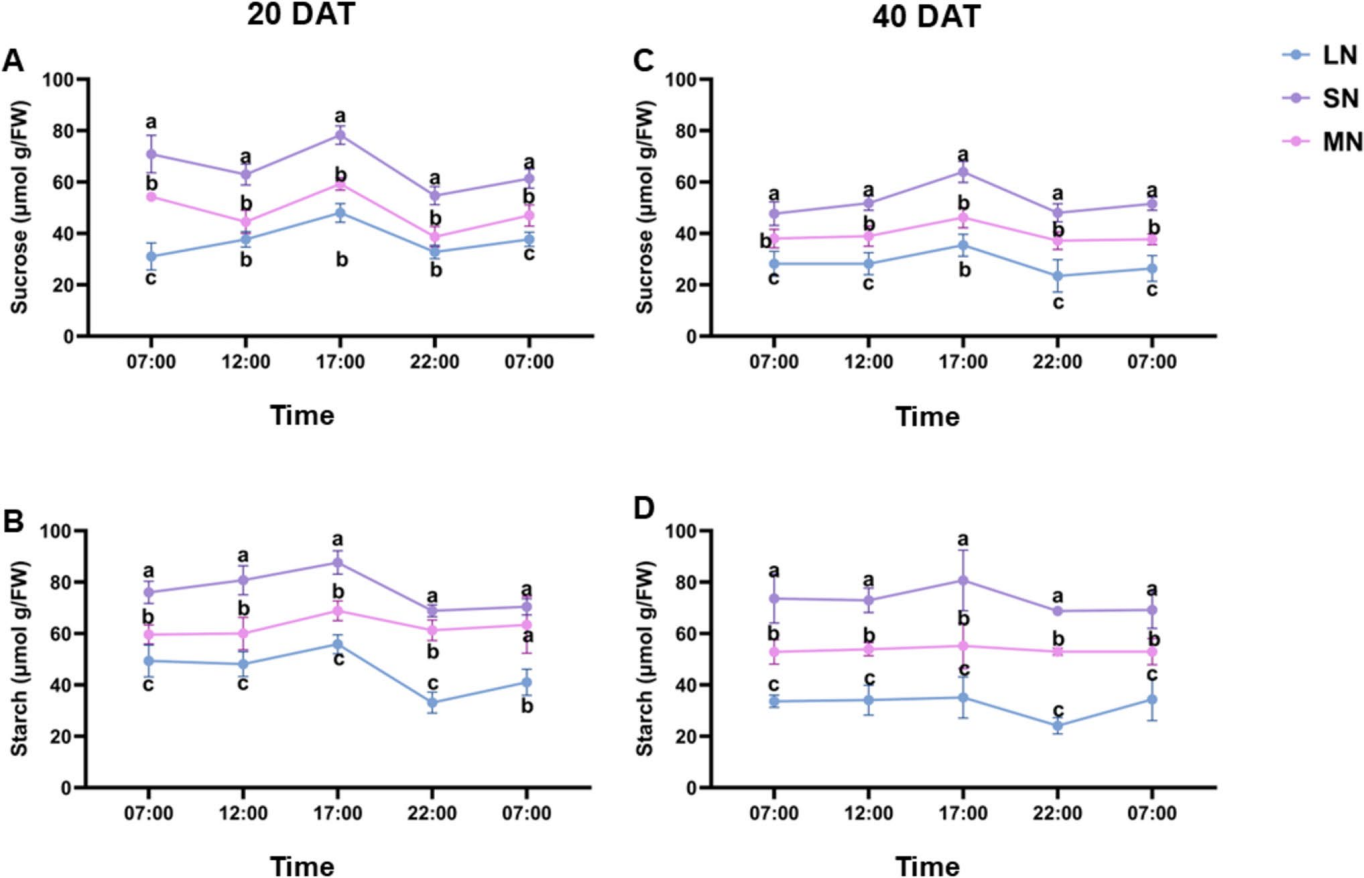
Diurnal changes in leaf sucrose and starch content under different N treatment levels. sucrose content at 20 days after transfer (DAT) (**A**), starch content at 20 DAT (**B**), sucrose content at 20 DAT (**C**) and starch content at 40 DAT (**D**). Samples were collected at five time points: 7:00, 12:00, 17:00, 22:00, and 7:00 on the following day. Data points represent the mean ± standard error (SE) of six independent biological replicates (*n* = 6). *, **, ***, ****, and ns denote significance at *P* ≤ 0.05, 0.01, 0.001, 0.0001, and not significant, respectively. Statistical significance was determined using two-way analysis of variance (ANOVA), followed by Tukey’s HSD post-hoc test. FW, fresh weight; LN, low nitrogen (1 mM NO₃⁻); MN, medium nitrogen (2 mM NO₃⁻); SN, nitrogen level supplementation (1 mM NO₃⁻ → 1 mM NO₃⁻)

The spatial distribution of sucrose and starch across different maize tissues, including the upper, middle, and basal leaves, the leaf sheath, and the roots, was examined under SN treatment (Fig. 8). Sucrose and starch showed distinct pattern of accumulation in all treatment groups, with SN plants showing a higher sucrose levels across all maize tissue, following by LN with optimum rate of accumulation and LN exhibiting the lowest sucrose and starch accumulation in all tissues (Fig. 8A-D). At 22:00, sucrose levels were elevated in the upper, middle, and basal leaves, as well as in the leaf sheath and roots, with SN-treated plants showed significantly (*P* ≤ 0.05) higher starch levels by the end of the day and LN plants exhibiting the lowest rate of accumulation of these metabolites (Fig. 8B, D). By 07:00 the next morning, SN-treated plants retained higher residual sucrose levels in the upper and basal leaves and the leaf sheath, with concentrations comparable to those recorded at 22:00 (Fig. 8A, C). Additionally, SN-treated plants accumulated higher starch levels in the leaves by 22:00 and maintained elevated starch concentrations following overnight remobilization. Starch content in the roots (sink tissues) was relatively higher and exhibited significant (*P* ≤ 0.05) variation among treatment groups at both 22:00 and 07:00 (Fig. 8B–D).

## Discussion

Maize growth and development is highly dependent on N. Our preliminary experiment revealed that varying N levels distinctly influenced seedling growth, photosynthesis, and C fixation. While high N treatment (10 mM NO₃⁻) increased shoot, root, and total biomass, low N (1 mM NO₃⁻) significantly elevated sucrose, soluble sugars, and starch levels in both leaves and roots. Interestingly, plants grown under moderate N (2 mM NO₃⁻) exhibited enhanced growth and photosynthetic activity, along with increased sugar and starch accumulation (Amoah et al. 2025) (Table S2). Recently, we demonstrated that switching the N source from NO₃⁻ to NH₄⁺ not only promoted shoot and root growth but also significantly increased carbon accumulation, thereby enhancing photosynthetic activity in maize seedlings. This effect was particularly pronounced in N form substitution (NFS) plants compared to those exposed to sole NO₃⁻ or NH₄⁺ treatments (Amoah and Kaiser 2025). However, the mechanistic basis underlying N level supplementation and its role in improving plant performance has not been explored. Given the growing attention on optimizing N application strategies, we hypothesize that N level supplementation may enhance plant growth beyond what is observed under static or fixed N treatments.

### N availability impacted phenotype performance, growth and photosynthesis

Maize seedlings showed distinct physiological and developmental response under varying N treatments (LN, SN and MN), each contributing uniquely to our understanding of the role of N in maize growth. LN significantly inhibited both shoot and root development (Fig. 2A-D and S1A-B) and resulted in smaller cobs by 40 DAT (Fig. S1A), demonstrating the drastic impact of low N availability on maize growth and yield (Amoah et al. 2025). This was further reflected in a pronounced reduction in Pn and chlorophyll content under LN (Fig. 2E-F), aligning with previous findings that N deficiency compromises photosynthetic efficiency and overall plant development (Zhao et al. 2020; Amoah and Kaiser 2025). In contrast, MN treatment showed a more distinctive and beneficial impact. Specifically, at 20 DAT, MN plant exhibited enhanced shoot and overall biomass accumulation, demonstrating a stimulatory role of moderate N availability during vegetative growth (Zhao et al. 2020). By 40 DAT, although shoot and root biomass increases were statistically insignificant, MN plants maintained higher root and total biomass, further suggesting that MN may supports maize root and overall plant development during the early reproductive (R1) stages (Amoah et al. 2025). This distinction is crucial, as it highlights MN as a potentially optimal N strategy for balancing vegetative and reproductive growth, differentiating it from LN and SN treatment (Zhou et al. 2023). Additionally, R/S ratio remained unchanged across all N treatments and growth stages, indicating that N availability modulates biomass accumulation more than biomass partitioning. Leaf Pn was higher across all treatments at 20 DAT but declined by 40 DAT, reflecting a general developmental pattern rather than treatment specific effects (Gu et al. 2017).

### N treatments distinctly enhanced amin acid and protein accumulation, thereby influencing N assimilation

N availability plays a crucial role in modulating plant N status, protein synthesis and amino acid metabolism (Dellero 2020). In this study, total N, amino acid and protein contents increased at 20 DAT and decline at 40 DAT across all treatment (Fig. 3A-F), demonstrating a developmental shift in N utilisation as plants transitioned from vegetative (V6) to reproductive (R1) stages. This dynamic change aligns with previous studies suggesting that N assimilation and protein synthesis are most active during early vegetative growth, followed by redistribution of N to reproductive organs (Masclaux-Daubresse et al. 2010; Xu et al. 2012; Kong et al. 2016). SN plants consistently showed the highest total N, protein and amino acid levels at both stages compared to plants under LN and MN treatments. This sustained N accumulation in SN plants may have contributed to enhanced photosynthetic performance and biomass production (Fig. 2), as highlighted in previous studies (Kant et al. 2010; Plett et al. 2016; Xu et al. 2012). The higher N concentrations indicate efficient N accumulation and assimilation, supporting enhanced metabolic activity at both growth stages. Similarly, SN plants exhibited higher protein and amino acid levels in the leaves and root at both stages, reinforcing the link between N availability and protein biosynthesis (Perchlik and Tegeder 2018). The protein and amino acid contents in the leaves significantly varied among treatments at both stages (Fig. 3C, E), indicating that N supply influences foliar metabolic capacity. In contrast, amino acid and protein differed significantly among treatments only at 40 DAT (Fig. 3D, F), demonstrating that root metabolic response is constrained by prolonged N deficiency, which aligns with previous findings that N deficiency reduces amino acid pools and protein synthesis in root tissues (Yang et al. 2020; Lai et al. 2023). The decline in total N, amino acid and protein at 40 DAT across all treatments may indicate a developmental reallocation of resources towards reproductive structure or a downregulation of N assimilation pathways, as plant growth slows (Bertheloot et al. 2008). Additionally, the lower levels of these N-related metabolites in LN plants highlights the detrimental effect of N deficiency on both structural and metabolic development, impairing the yield potential and stress resilience (Fig. S1A) (Schlüter et al. 2012).

### N availability shapes metabolic and transcriptional responses in maize

N availability significantly influenced the accumulation of sugars and starch, as well as the activity and expression of key metabolizing enzymes and genes in maize seedlings. The dynamic changes observed across developmental stages and N treatments underscore the enhanced coordination between N status and C metabolism. At 20 DAT, fructose and glucose levels were higher in both the leaves and roots and significantly differed among treatments (Fig. S2A-D). These early increases may reflect enhanced metabolic activity during vegetative growth, when energy demands are relatively high (Lazare et al. 2021). However, the fructose and glucose levels declined significantly (*P* ≤ 0.05) by 40 DAT, indicating a shift in C partitioning to support transition to reproductive development. Contrary to the glucose and fructose level, sucrose, soluble sugar and starch concentrations were lower at 20 DAT but increased significantly at 40 DAT, and this increase was pronounced in SN plants (Fig. 4A-F). This pattern demonstrates a developmental reprograming of C storage, with SN plants exhibiting increased capacity for carbohydrates accumulation, which may be due to improved assimilation and photosynthetic efficiency, as highlighted in previous studies (Santiago and Tegeder 2017; López-González et al. 2022).

The activities of sugar-metabolizing enzymes (SPS, SuSy, CINV, and VINV) were influenced by both N availability and growth stage. Enzyme activities peaked at 20 DAT and declined by 40 DAT, with SN plants maintaining significantly higher levels, MN plants showing intermediate levels, and LN plants exhibiting the lowest activities in both leaves and roots (Figs. 5A–D, S3A–D). Interestingly, the root SuSy activity did not vary among treatments at 40 DAT, indicating tissue-specific modulation under prolonged N deficiency associated stress (Gordon et al. 1997). Similarly, the activities of starch-metabolizing enzymes, such as SS, AGPase, AMY and BAM, exhibited a similar pattern, with SN plants showing consistently upregulated activities compared to plants under MN and LN treatments, which showed moderate and lowest activities (Figs. 5E-F, S3E-F),), indicating that N deficiency suppresses C metabolism, a pattern that aligns with previous findings showing that n stress significantly disrupts sugar and starch metabolic pathways in maize (Song et al. 2023). Moreover, the gene expression analysis supported these biochemical trends, with sugar metabolism-related (*ZmSPS1*, *ZmSuSy1*, *ZmSUT2*, *ZmSUC2*, *ZmSWEET14*, *ZmSTP2*, *ZmCINV1*, *ZmVINV1*) and starch metabolism-related genes (*ZmSS1*, *ZmAGPase1*, *ZmAMY1*, *ZmBAM1*) exhibiting higher upregulation at 20 DAT than 40 DAT (Figs. 6 and 7A-H). SN plants consistently exhibited the highest transcript abundance in both leaves and roots, indicating a strong transcriptional response to optimal N supply (Figs. 6-7A-H and S4A-B). Typically, *ZmSWEET14*, expression reached up to 5.2- and 3.8-fold in the leaves of LN and MN plants, while *ZmSUC2* expression peaked at 7.8-fold in the root of SN plants. These data suggest that SN enhanced sugar transport and mechanism, supporting robust growth and development in maize.

The coordinated upregulation of sugar and starch metabolism-related enzymes and genes in plants grown under SN treatment reflects synergistic interaction between C and N pathways. Enhanced N availability promotes photosynthetic activity, resulting in increased C fixation and storage (Gao et al. 2018; Li et al. 2020; Mu and Chen 2021). Conversely, LN impaired these processes, causing a reduction and sugar accumulation, lower sugar and starch metabolizing enzymes activities, and downregulation of their-related gene expression. These dynamic changes may compromise energy supply and stress adaptation, which ultimately the yield (Fig. 1A) of maize, as highlighted in previous studies (Amoah et al. 2025; Amoah and Kaiser 2025). Collectively, the study findings demonstrate that N availability modulates N assimilation and C metabolism through enzymatic and transcriptional control. The superior performance of SN than LN and MN plants across all indicators examined underscores the importance of adequate N supply in optimizing metabolic capacity and developmental progression in maize (Yue et al. 2021; Meng et al. 2024).

### Diurnal regulation of sucrose and starch reflects N-dependent C dynamics

Diel patterns of sucrose and starch accumulation revealed consistent rhythms across all N treatments, with levels rising after midday, peaking at 17:00, declining by 22:00, and recovering by 07:00 (Figs. 8A–D). These trends were not immediate responses to fertigation but rather reflected endogenous regulation, which may be driven by circadian rhythms and photoperiod cues that modulate C allocation independently of short-term nutrient fluctuations (Graf et al. 2010; Xiao et al. 2024; Amoah and Kaiser 2025). SN-treated plants consistently exhibited the highest sucrose and starch levels throughout the day and retained elevated pools overnight. This indicates enhanced photosynthetic capacity, efficient C storage, and stable source–sink coordination under sufficient N supply. N availability is known to stimulate chloroplast development and enzymatic activity involved in carbohydrate biosynthesis, thereby promoting C assimilation and retention (Slewinski and Braun 2010). MN plants maintained moderate levels, indicating a balanced C profile with functional rhythmic regulation. Although not as elevated as SN, MN plants showed adequate retention and diel consistency, reflecting moderate N support for carbon metabolism and rhythmic control (Amoah and Kaiser 2025). Additionally, LN plants displayed the lowest carbohydrate levels and reduced overnight retention, suggesting impaired C assimilation and weakened sink strength under N deficiency. N deficiency has been shown to restrict photosynthetic efficiency and reduce sugar transport capacity, thereby limiting carbohydrate accumulation (Scheible et al. 2004; Nunes-Nesi et al. 2010). Despite this, the diel rhythm remained intact, suggesting that the timing of accumulation is regulated by circadian regulatory networks rather than nutrient status alone. N availability modulated the magnitude of sucrose and starch accumulation, while circadian regulation preserved the timing of diel fluctuations. These findings highlight the interplay between nutrient supply and internal metabolic rhythms, offering insights into adaptive C/N coordination in maize and broader implications for improving nutrient use efficiency in crop systems.

### Spatial distribution of sucrose and starch reflects N dependent C allocation strategies

**Fig. 9:**
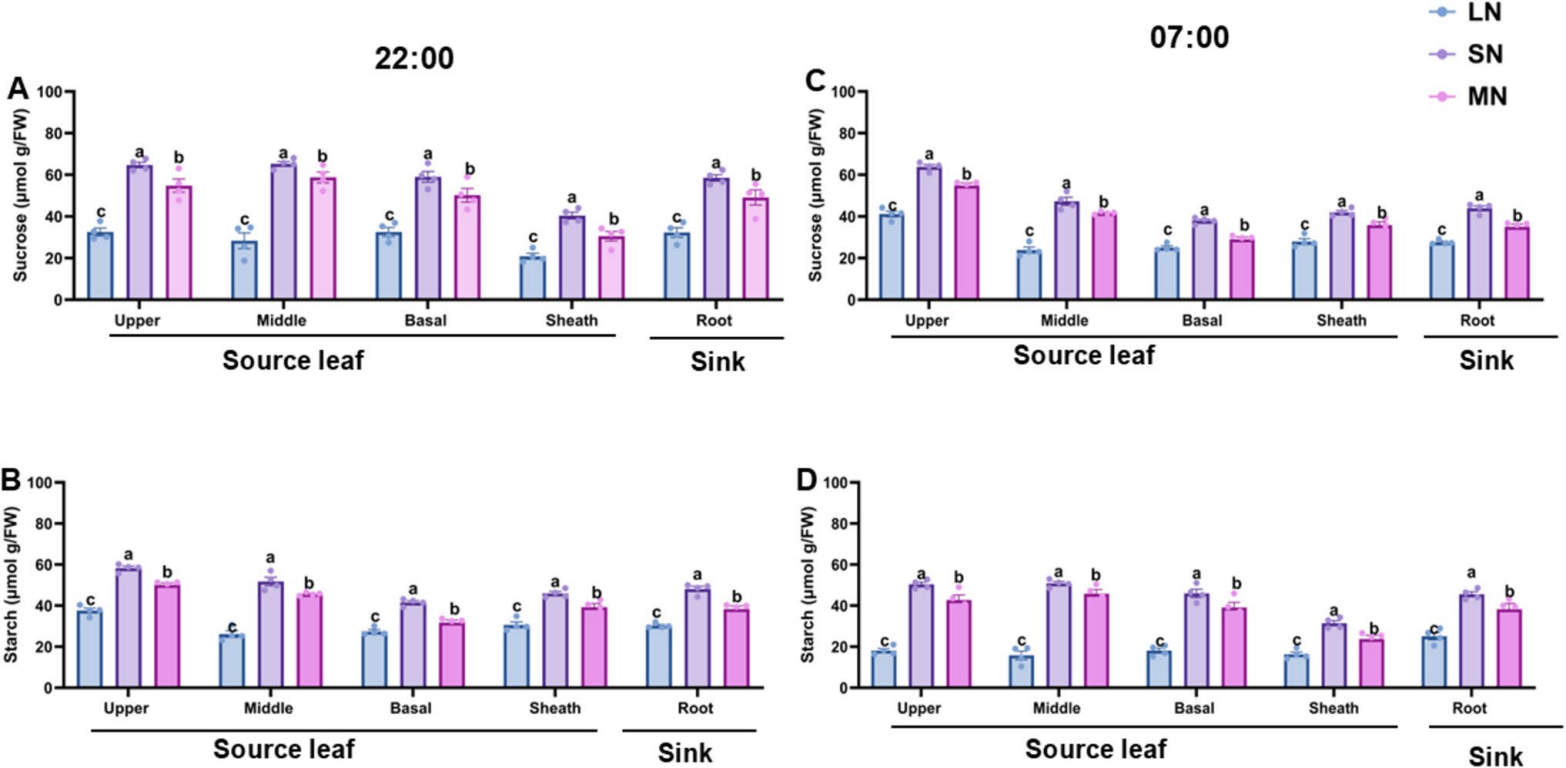
Spatial and temporal analysis of sucrose and starch content in different maize tissues at 40 DAT. Leaf sucrose (**A**) and starch (**B**) levels at 22:00, and leaf sucrose (**C**) and starch (**D**) levels at 07:00, measured under different nitrogen treatment levels. Data points represent the mean ± standard error (SE) of six independent biological replicates (*n* = 6). *, **, ***, ****, and ns denote significance at *P* ≤ 0.05, 0.01, 0.001, 0.0001, and not significant, respectively. Statistical significance was determined using two-way analysis of variance (ANOVA), followed by Tukey’s HSD post-hoc test. FW, fresh weight; LN, low nitrogen (1 mM NO₃⁻); MN, medium nitrogen (2 mM NO₃⁻); SN, nitrogen level supplementation (1 mM NO₃⁻ → 1 mM NO₃⁻)

The spatial distribution of sucrose and starch in maize tissues, such as upper, middle, and basal leaves, leaf sheath, and roots identifies distinct C allocation strategies shaped by N availability (Fig. 9). These patterns provide valuable insight into how maize seedlings adapt their metabolic responses to optimize growth under varying N conditions (Amoah and Kaiser 2025). Sucrose and starch concentrations varied significantly across treatments at both 22:00 and 07:00, underscoring the dynamic nature of C allocation and the pivotal role of roots as metabolic sinks under SN conditions (Zhao et al. 2020). The pronounced accumulation of starch in root tissues suggests that SN availability strengthens sink demand, thereby promoting nutrient uptake and supporting sustained growth. Tissue-specific C distribution under SN conditions appears to reflect a coordinated strategy to maintain metabolic homeostasis, which includes the retention of sucrose and starch in leaves and the leaf sheath as transient storage pools, which are remobilized overnight to meet early morning energy demands. The roots function as stable C sinks, facilitating nutrient acquisition and signalling integration (Xiao et al. 2024; Amoah et al. 2025). These spatial patterns suggest that maize seedlings under SN conditions prioritize both immediate metabolic needs and long-term resource acquisition through strategic C partitioning. Understanding the regulatory mechanisms underlying these spatial dynamics, particularly the influence of diurnal rhythms on source–sink transitions, will provide valuable information for crop improvement. Furthermore, insights into tissue-specific C allocation can inform targeted breeding and genetic engineering strategies aimed at enhancing plant performance under fluctuating N environments. Future research will focus on elucidating the molecular and enzymatic pathways that modulate C remobilization, such as the functions of sucrose transporters, starch metabolism enzymes, and circadian regulators.

## Conclusion

**Fig. 10:**
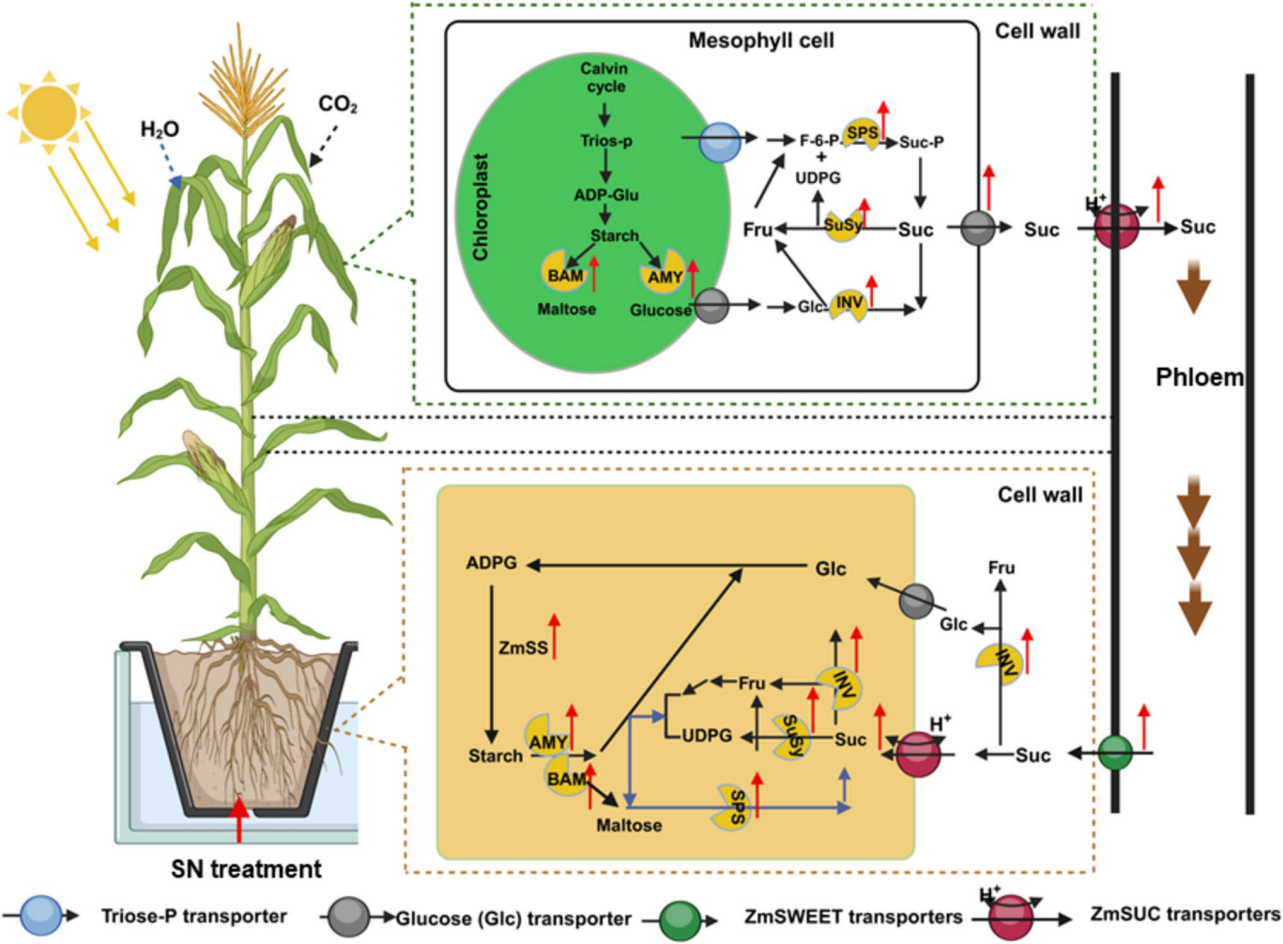
A conceptual model illustrating sugar metabolism in the maize inbred line TX-40 J under supplemental nitrogen (SN) condition. The SN treatment triggers a sugar-mediated cascade response that enhances carbon accumulation. It alters the expression of key regulatory genes and modulates the activities of sugar-metabolizing enzymes, thereby influencing sugar accumulation. Furthermore, SN activates the transcription of sugar transporter genes, facilitating the redistribution of sugars to support adaptive responses under SN condition. Upregulated genes and enzymes are represented by upward red arrows

The study demonstrated that N availability influences C allocation and metabolism in maize. SN treatment significantly promoted shoot and root development, biomass accumulation, and overall phenotypic performance, alongside enhanced photosynthetic activity compared to plants grown under LN and MN treatment. These improvements were closely linked to the regulation of sugar metabolism, allocation, and transport (Fig. 10). Under SN conditions, the activities of key sucrose and starch-metabolizing enzymes, such as SPS, SuSy, CINV, VINV, SS, AGPase, AMY, and BAM, were markedly elevated. Correspondingly, the expression of their associated genes (*ZmSPS1*, *ZmSuSy1*, *ZmCINV1*, *ZmVINV1*, *ZmSS1*, *ZmAGPase1*, *ZmAMY1*, and *ZmBAM1*) was upregulated in both leaves and roots, enhancing carbohydrate turnover and utilization efficiency. Similarly, SN treatment significantly increased the expression of sucrose transporter genes, *ZmSWEET14*, *ZmSUC2*, *ZmSTP2*, and *ZmSUT2*, facilitating efficient phloem loading and unloading and promoting sucrose translocation from source leaves to sink tissues. These coordinated molecular and physiological responses underscore SN as a promising strategy for improving NUE and advancing sustainable agricultural practices. To maximize the translational impact of these findings, future research will explore the broader applicability of SN-enhanced NUE across diverse crop species and agroecosystems. Field trials under varying soil types, climatic conditions, and management regimes will be essential to validate these outcomes in real-world settings. While this study focused on the high-performing inbred maize line TX-40 J, evaluating a broader range of genotypes will provide deeper insights into genetic variability in N responsiveness. Expanding the range of N concentrations and incorporating additional control treatments will further clarify the physiological thresholds and regulatory mechanisms underlying plant responses to N availability. Moreover, integrating transcriptomic and metabolomic approaches will enable precise characterization of gene function across developmental stages and treatment conditions, offering a more comprehensive understanding of the molecular networks modulating C/N interactions in maize.

## Supplementary Information

Below is the link to the electronic supplementary material.

ESM1 (DOCX 1.08 MB)

ESM2 (DOCX 18.6 KB)

## Data Availability

Data is contained in the manuscript.
