# Correction to: Supplemental nitrogen induces robust physiological and molecular adaptations by enhancing carbon metabolism in maize

**DOI:** 10.1007/s00709-025-02121-6

**Published:** 2025-10-10

**Authors:** Joseph N. Amoah, Claudia Keitel, Brent N. Kaiser

**Affiliations:** School of Life and Environmental Sciences, University of Sydney, 380 Werombi Road, Brownlow Hill, Camden, NSW 2570 Australia

**Correction to: Protoplasma**

10.1007/s00709-025-02116-3

During the submission process, a limited number of primer sequences were incorrectly included in Table S1 and the target gene LOC identifier was not included. To correct this, we wish to update the supplementary file with the appropriate gene names and corresponding NCBI LOC identifiers to improve clarity and facilitate reproducibility for readers and researchers. This update does not affect the manuscript’s content, results, or conclusions, but serves as an annotation enhancement. We are terribly sorry for this mistake and the impact the incorrect sequences may have had to other research groups, readers and importantly the journal.

