# Supplementary material for: Supplemental nitrogen induces robust physiological and molecular adaptations by enhancing carbon metabolism in maize
Source: Protoplasma. 2025 Sep 26;263(2):403–20. doi: 10.1007/s00709-025-02116-3 (PMC12945941; doi:10.1007/s00709-025-02116-3)
Supplement: Supplementary file 1 — (DOCX 1.08 MB) [file 709_2025_2116_MOESM1_ESM.docx]

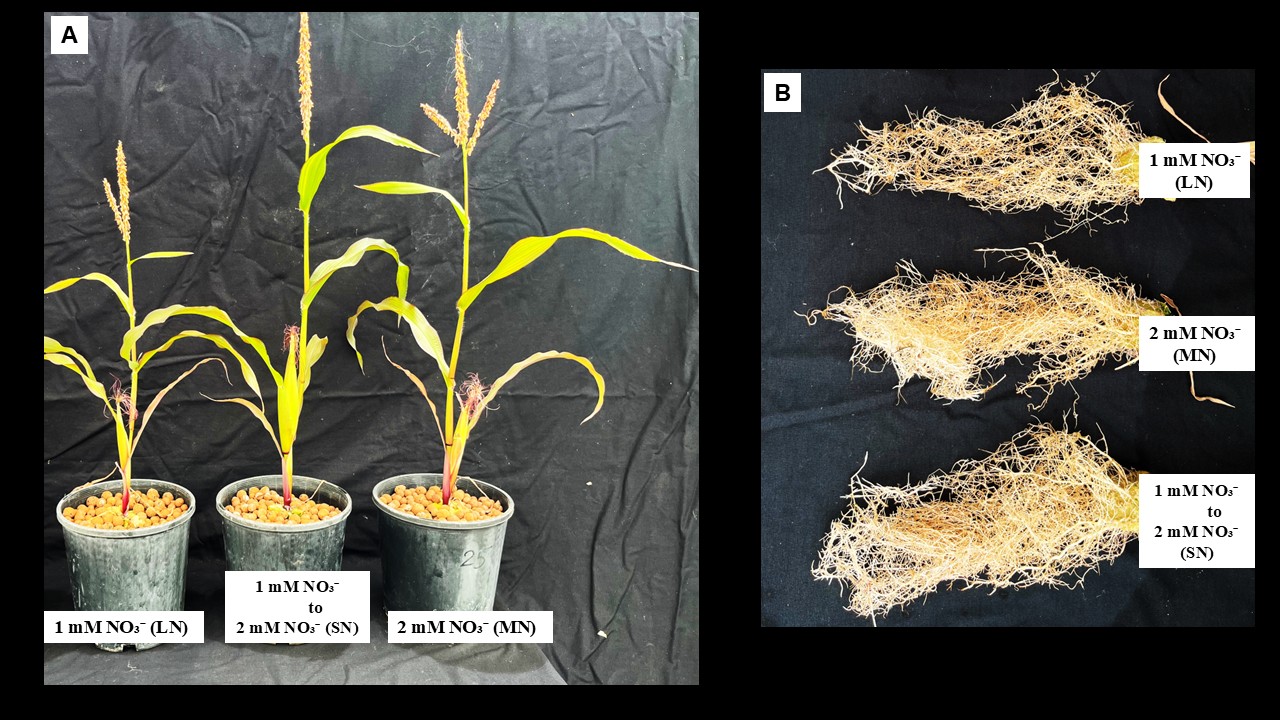


**Fig. S1** Phenotypic response of the maize inbred line TX-40J to different nitrogen supplementation (SN) treatment. Representative images of shoot (A) and root (B) morphological changes at 40 days after nitrogen treatments (DAT).


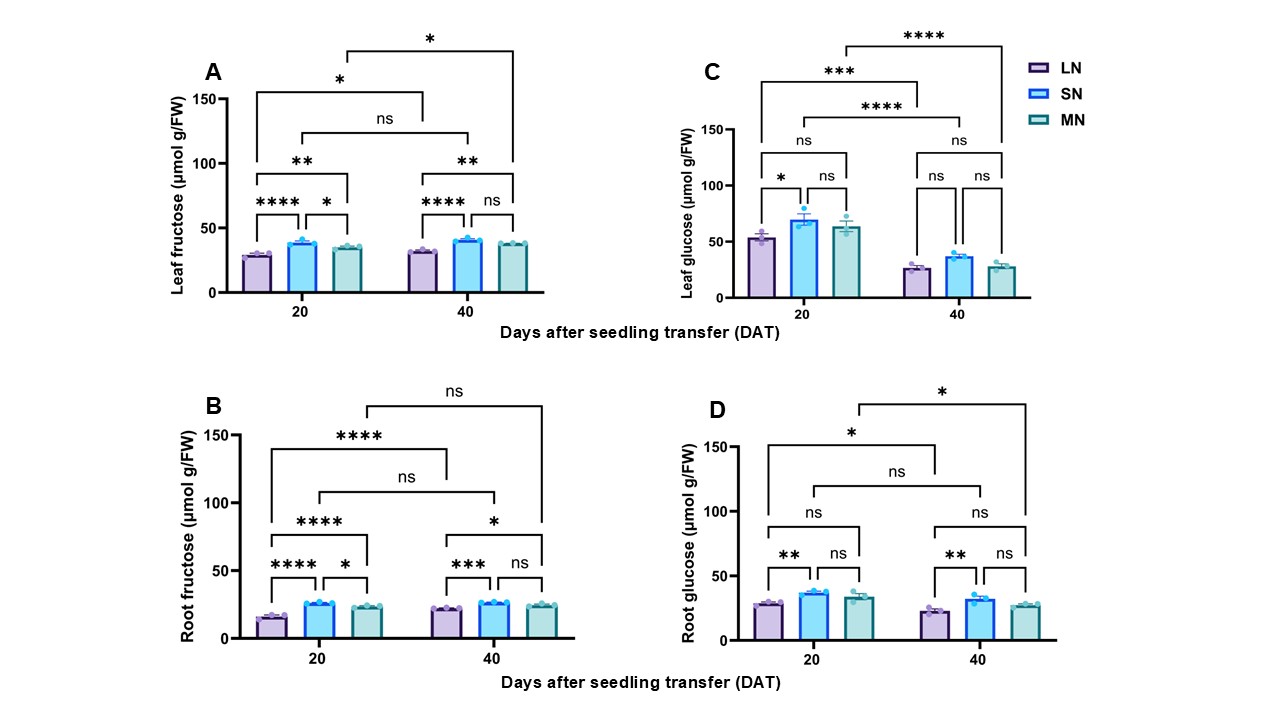


**Fig S2** Leaf (**A**) and root (**B**) fructose, and leaf (**C**) and root (**D**) glucose content under different nitrogen levels. Data points represent the mean ± standard error (SE) of six independent biological replicates (n = 6). *, **, ***, ****, and ns denote significance at *P* ≤ 0.05, 0.01, 0.001, 0.0001, and not significant, respectively. Statistical significance was determined using two-way analysis of variance (ANOVA), followed by Tukey’s HSD post-hoc test. FW, fresh weight; LN, low nitrogen (1 mM NO₃⁻); MN, medium nitrogen (2 mM NO₃⁻); SN, nitrogen level supplementation (1 mM NO₃⁻ → 1 mM NO₃⁻).


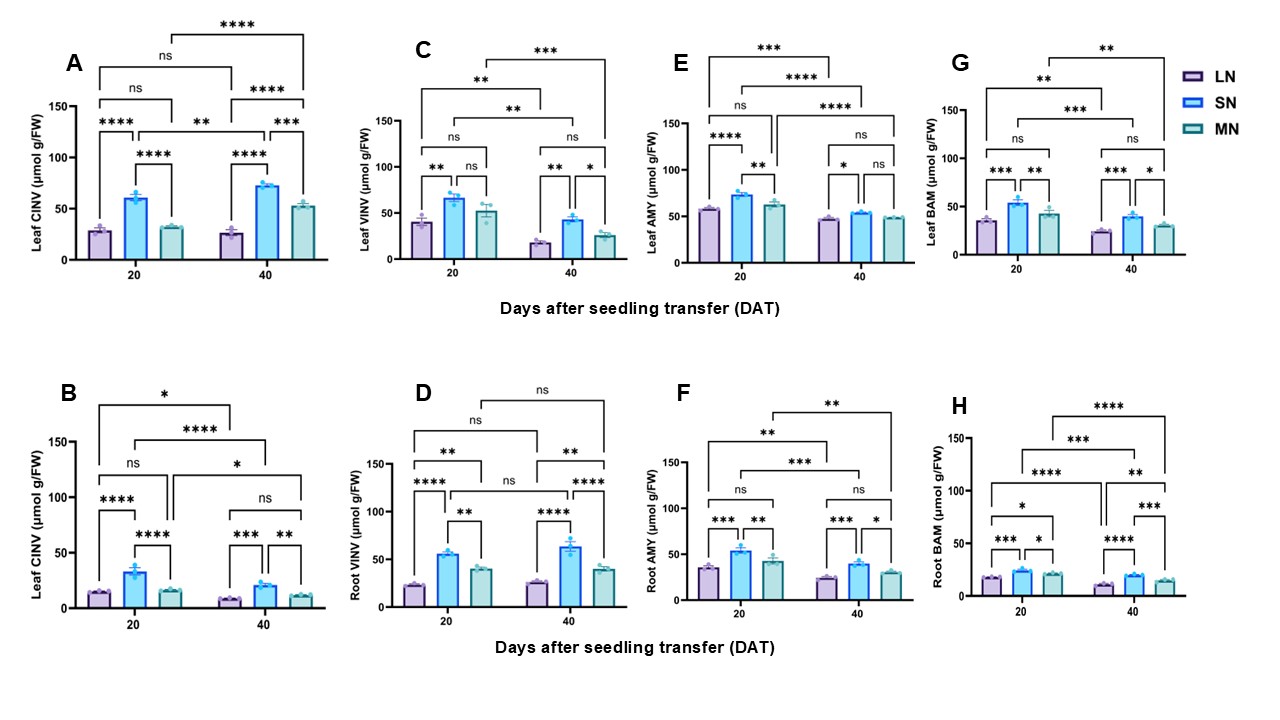


**Fig. S3** Leaf cytoplasmic invertase, vacuolar invertase, α-amylase, and β-amylase activities in leaves (**A**, **C**, **E**, and **G**) and roots (**B**, **D**, **F**, and **H**) under varying nitrogen levels. Data points represent the mean ± standard error (SE) of six independent biological replicates (n = 6). *, **, ***, ****, and ns denote significance at *P* ≤ 0.05, 0.01, 0.001, 0.0001, and not significant, respectively. Statistical significance was determined using two-way analysis of variance (ANOVA), followed by Tukey’s HSD post-hoc test. FW, fresh weight; LN, low nitrogen (1 mM NO₃⁻); MN, medium nitrogen (2 mM NO₃⁻); SN, nitrogen level supplementation (1 mM NO₃⁻ → 1 mM NO₃⁻).


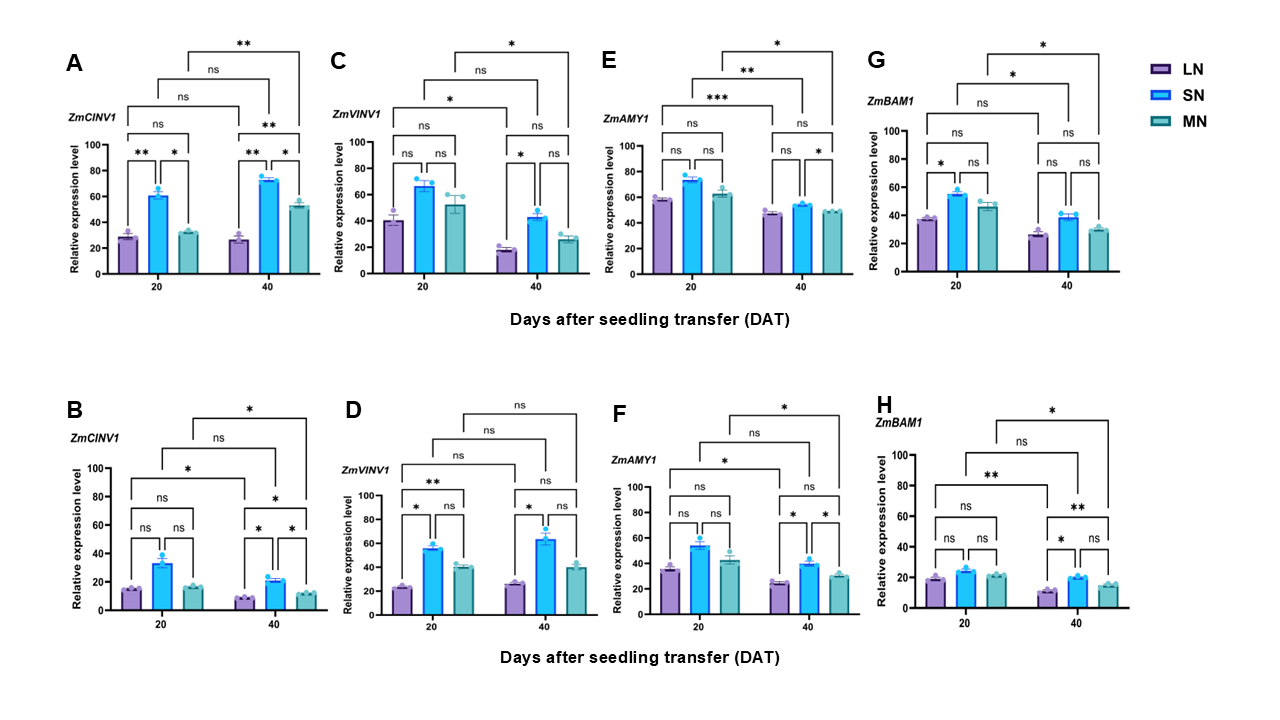


**Fig. S4** Expression patterns of sugar and starch metabolism enzymes under different N levels. Relative expression levels of *ZmCINV1*, *ZmVINV1*, *ZmAMY1* and *ZmBAM1* in the leaf (**A**, **C**, **E** and **G**) and root (**B**, **D**, **F** and **H**) of the maize inbred line TX-40J. Data points represent the mean ± standard error (SE) of six independent biological replicates (n = 6). *, **, ***, ****, and ns denote significance at *P* ≤ 0.05, 0.01, 0.001, 0.0001, and not significant, respectively. Statistical significance was determined using two-way analysis of variance (ANOVA), followed by Tukey’s HSD post-hoc test. FW, fresh weight; LN, low nitrogen (1 mM NO₃⁻); MN, medium nitrogen (2 mM NO₃⁻); SN, nitrogen level supplementation (1 mM NO₃⁻ → 1 mM NO₃⁻).


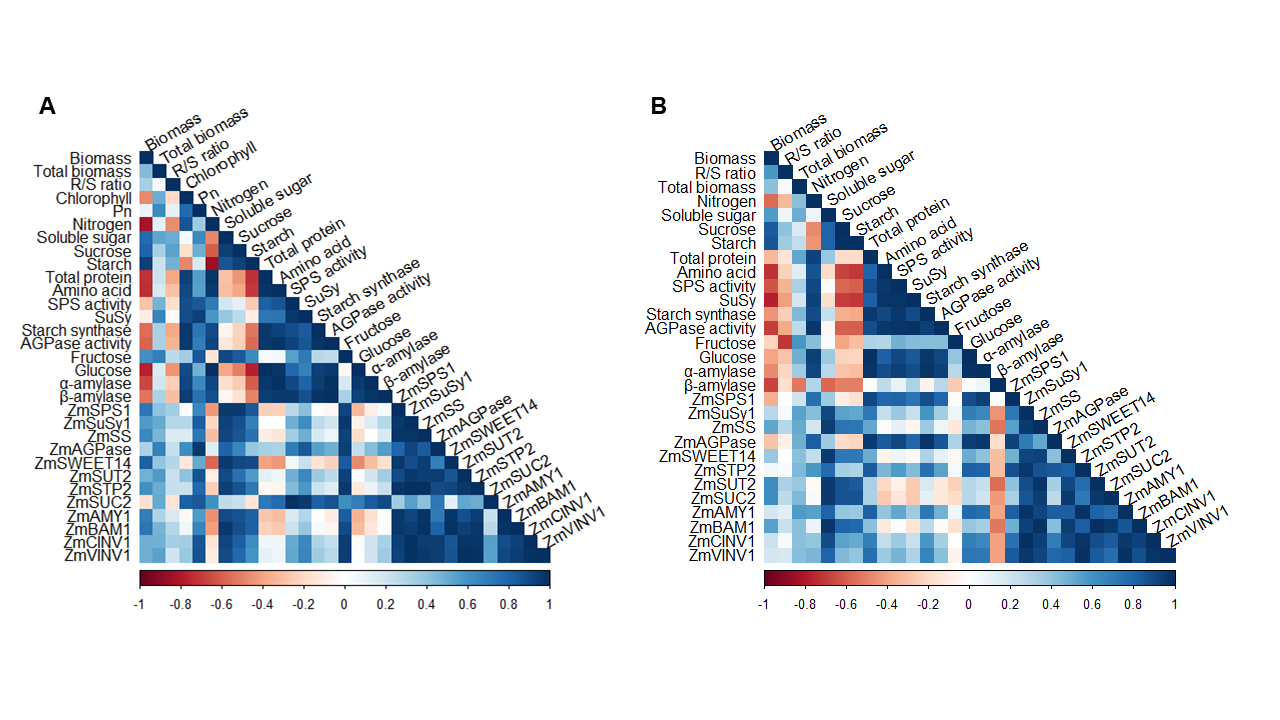


**Fig. S5** Pearson’s correlation plot of physio-biochemical and molecular indicators investigated in the leaf and root of maize inbred line TX-40J plants grown under nitrogen level supplementation (SN). The correlation plot was generated using the mean values of all treatment groups with GraphPad Prism (v10.1). Heatmap colours ranged from blue (high correlation) to red (low correlation).
