# Supplementary material for: Supplemental nitrogen induces robust physiological and molecular adaptations by enhancing carbon metabolism in maize
Source: Protoplasma. 2025 Sep 26;263(2):403–20. doi: 10.1007/s00709-025-02116-3 (PMC12945941; doi:10.1007/s00709-025-02116-3)
Supplement: Supplementary file 2 — (DOCX 18.6 KB) [file 709_2025_2116_MOESM2_ESM.docx]

**Table S1. List of genes studied**

| Gene name | NCBI LOCs |
| --- | --- |
| *ZmSuSy1*  *ZmSUC2*  *ZmSWEET14*  *ZmActin*  *ZmUBQc*  *ZmSPS1*  *ZmAGPase1*  *ZmSUT2*  *ZmSTP2*  *ZmSS1* | LOC542247  LOC541615  LOC100282708  LOC100282267  LOC541665  LOC542711  LOC542737  LOC100280251  LOC100273083  LOC541669 |

**Table S2:** Effect of different nitrogen (N) levels on maize growth and carbon metabolism at 20 days after seedling transfer

| Trait/(plant) |  | Shoot/leaf |  |  | Root |  |
| --- | --- | --- | --- | --- | --- | --- |
|  | **1 mM NO_3_^-^** | **2 mM NO_3_^-^** | **10 mM NO_3_^-^** | **1 mM NO_3_^-^** | **2 mM NO_3_^-^** | **10 mM NO_3_^-^** |
| Biomass  Total biomass  Root: shoot ratio  Sucrose  Soluble sugar  Starch | 0.236 ± 0.02c  0.374 ± 0.01c  0.59 ± 0.06a  98.566 ± 0.73a  92.610 ± 1.69a  86.633 ± 1.63a | 0.372 ± 0.03b  0.460 ± 0.03b  0.237 ± 0.03b  90.410 ± 2.11b  84.083 ± 1.57b  77.766 ± 1.03b | 0.521 ± 0.02a  0.619 ± 0.02a  0.188 ± 0.02c  84.033 ± 0.64c  75.583 ± 0.34c  67.133 ± 1.21c | 0.1386 ± 0.004a  0.374 ± 0.01c  0.59 ± 0.06a  29.511 ± 1.04a  31.713 ± 1.19a  33.916 ± 1.36b | 0.0876 ± 0.02c  0.460 ± 0.03b  0.237 ± 0.03b  23.388 ± 0.62b  32.361 ± 0.50a  41.33 ± 0.96a | 0.0976 ± 0.01a  0.619 ± 0.02a  0.188 ± 0.02c  20.077 ± 0.29c  25.097 ± 0.71b  30.116 ± 1.23c |

Data points represent the mean ± error (SE) of six independent biological replicates (n = 6). Different letters above the error bars indicate statistically significant differences at p ≤ 0.05. Abbreviations: DAT – days after seedling transfer; FW – fresh weight; DW – dry weight.
